# CUR5g, a novel autophagy inhibitor, exhibits potent synergistic anticancer effects with cisplatin against non-small-cell lung cancer

**DOI:** 10.1038/s41420-022-01217-9

**Published:** 2022-10-31

**Authors:** Jingxuan Chen, Yunpeng Shen, Bowen Wu, Peichang Yang, Gangchun Sun, Xiaoting Liu, Pengfei Qiang, Yamei Gao, Fangfang Sha, Zirui Li, Lu Zhang

**Affiliations:** 1 College of Bioengineering, Henan University of Technology, Lianhua Street, Zhengzhou, 450001 China; 2 College of Chemistry and Chemical Engineering, Henan University of Technology, Lianhua Street, Zhengzhou, 450001 China

**Keywords:** Macroautophagy, Cancer

## Abstract

Autophagy, a highly conserved degradation process of eukaryotic cells, has been proven to be closely related to chemoresistance and metastasis of non-small-cell lung cancer (NSCLC). Autophagy inhibitors, such as chloroquine (CQ) and its derivative hydroxychloroquine (HCQ), has been shown to mediate anticancer effects in preclinical models, especially when combined with chemotherapy. However, the vast majority of autophagy inhibitors, including CQ and HCQ, actually disrupt lysosomal or/and possibly non-lysosomal processes other than autophagy. It is therefore of great significance to discover more specific autophagy inhibitors. In this study, after screening a series of curcumin derivatives synthesized in our laboratory, we found that (3E,5E)-1-methyl-3-(4-hydroxybenzylidene)-5-(3-indolymethylene)-piperidine-4-one (CUR5g) selectively inhibited autophagosome degradation in cancer cells by blocking autophagosome-lysosome fusion. CUR5g did not affect the lysosomal pH and proteolytic function, nor did it disturb cytoskeleton. CUR5g blocked the recruitment of STX17, a soluble N-ethylmaleimide-sensitive factor attachment protein receptor (SNARE) protein, to autophagosomes *via* a UVRAG-dependent mechanism, resulting in the inability of autophagosomes to fuse with lysosomes. CUR5g alone did not induce apoptosis and necrosis of A549 cells, but significantly inhibited the mobility and colony formation of A549 cells. More excitingly, CUR5g showed no obvious toxicity to normal HUVECs in vitro or mice in vivo. CUR5g enhances the cisplatin sensitivity of A549 cells and effectively inhibited autophagy in tumor tissues in vivo. Collectively, our study identified a new late-stage autophagy inhibitor and provided a novel option for NSCLC treatment, particular when combined with cisplatin.

## Introduction

Lung cancer, with an estimated 2.2 million new cancer cases and 1.8 million deaths in 2020, remains the leading cause of cancer deaths [1]. The overwhelming majority (approximately 85%) of lung cancer is non-small cell lung cancer (NSCLC), and more than 70% of cases are diagnosed at an advanced stage when surgery is not appropriate [2]. Even with successful surgical resection in early stage, a considerable number of patients develop local or distant recurrences [3]. Cisplatin-based combination have been recommended as the first-line treatments for NSCLC, but numerous undesirable effects including chemotherapy resistance and adverse reactions are still the vital bottlenecks which limit its curative efficiency in most NSCLC patients. Thus, it is of great importance to identify novel low-toxic agents that increase the sensitivity of NSCLC cells to cisplatin.

Autophagy supplies the demand for NSCLC cells survival under unfavorable conditions and helps them cope with threatening stressors, which has been considered as an important mechanism of chemotherapy resistance [4]. High levels of basal autophagy are prevalent in cisplatin-resistant cancer cells and are thought to confer survival benefits [4]. Increasing number of studies have demonstrated that block of autophagy effectively increased the sensitivity of NSCLC cells to cisplatin [5–7], thereby providing a reasonable basis for the combined use of autophagy inhibitors and cisplatin for NSCLS treatment.

Currently, chloroquine (CQ) and its derivative hydroxychloroquine (HCQ), which inhibit autophagosome degradation by limiting the acidification of lysosomes, are still the only clinically available autophagy inhibitors [8]. Several studies have shown that CQ or HCQ enhanced cisplatin-induced cytotoxicity in NSCLC cells [7, 9, 10], but there is still a lack of clinical data on the combination of CQ or HCQ and cisplatin in the treatment of NSCLC. Importantly, clinical studies showed that even at a high dose of 1200 mg/d, HCQ produces only modest autophagy inhibition in human tissues [11]. Although generally considered to be well tolerated in vivo, several side effects of HCQ and CQ have been reported, including gastrointestinal discomfort, retinopathy, cardiomyopathy, and so on [12]. The lack of potency in inhibiting autophagy and off-target toxicity of CQ and HCQ create a demand for the discovery of novel autophagy inhibitors that specifically target autophagy in cancer cells without interfering with other cellular processes and concurrently minimizing autophagy dysfunction in normal cells.

The vast majority of newly discovered autophagy inhibitors that inhibit tumor growth, either alone or in combination with chemoradiotherapy, are natural products or their derivatives by structural modifications, such as oblongifolin C, liensinine, elaiophylin, hederagenin, alpha-hederin, CA-5f, etc. [13–18]. Curcumin has been proved to be a naturally occurring autophagy modulator with anti-tumor activity, however, its reduced bioavailability in *vivo* severely limits its clinical implication [19]. Curcumin derivatization is an effective strategy to overcome such obstacle. More excitingly, some curcumin derivatives as newly identified autophagy inhibitors have been shown to selectively inhibit NSCLC without obvious cytotoxic influences on normal cells [18, 20].

In this study, we found that (3E,5E)-1-methyl-3-(4-hydroxybenzylidene)-5-(3-indolymethylene)-piperidine-4-one (CUR5g), a curcumin derivative, selectively induced autophagosome accumulation in cancer cells by blocking autophagosome-lysosome fusion. CUR5g blocked the recruitment of STX17 to autophagosomes *via* a UVRAG-dependent mechanism, resulting in the inability of autophagosomes to fuse with lysosomes. CUR5g exerts effectively anti-proliferative effects against NSCLC A549 cells. More excitingly, the combination use of CUR5g dramatically improved the anticancer effect of cisplatin against A549 cells both in vitro and in vivo.

## Results

### CUR5g selectively induces autophagosome accumulation in cancer cells

**Fig. 1:**
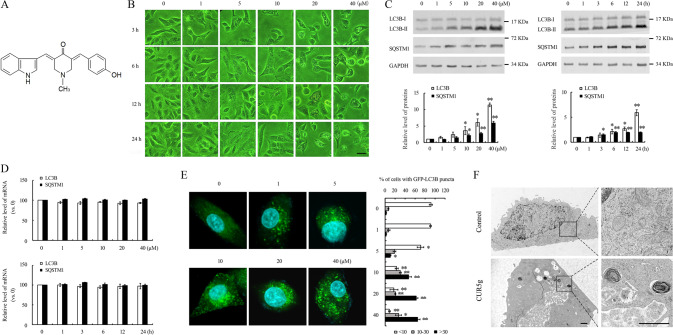
CUR5g modulates autophagy in A549 cells. **A** The chemical structure of CUR5g. The molecular formula of this compound is C_22_H_20_N_2_O_2_, and its molecular weight is 344.41 g/mol. **B** Representative microscopy photographs of A549 cells treated with various doses (0–40 μM) of CUR5g for 3–24 h. Scale bar = 40 μm. **C** Western blot analysis of LC3B-II and SQSTM1 levels in A549 cells treated with various doses (0–40 μM) of CUR5g for 24 h, or in the cells treated with 10 μM CUR5g for 0–24 h. GAPDH was used as a loading control. (*n* = 3; *p < 0.05, **p < 0.01 vs. 0). **D** qRT-PCR analysis of LC3B and SQSTM1 mRNA levels. **E** Representative fluorescence photographs of GFP-LC3B puncta in A549 cells treated with various doses (0–40 μM) of CUR5g for 24 h. Scale bar = 10 μm. Nuclei were stained with DAPI. Histogram shows quantification of the percentage of cells with GFP-LC3B puncta. (*n* = 3; *p < 0.05, **p < 0.01 vs. 0). **F** Representative transmission electron micrographs of A549 cells treated with DMSO or 10 μM CUR5g for 24 h. Boxed areas at left are enlarged at right. Scale bar = 1 μm.

This work used U87 cells with stable expression of GFP-LC3B fusion protein to screen various curcumin analogs to find new small-molecule inhibitors of autophagy. Among these analogs, CUR5g (Fig. 1A) induced extensive cytoplasmic vacuolization, and GFP-LC3B signal shifted from diffuse cytosolic staining to a punctate pattern outlining autophagosomes (Figs. 1B and S1), suggesting that CUR5g might regulate autophagy. As revealed by WB assay, CUR5g up-regulated LC3B-II and sequestosome 1 (SQSTM1) levels time- and dose-dependently (Fig. 1C). This increase was not the result of enhanced transcription, as mRNA expression of SQSTM1 and LC3B were not increased within CUR5g-exposed cells (Fig. 1D), suggesting that CUR5g might block autophagic flux rather than increase autophagosome formation.

Excitingly, CUR5g also induced SQSTM1 and LC3B-II accumulation in other human cancer cells (Fig. S2), but not in normal cells (Fig. S3). The punctuate distribution of GFP-LC3B was significantly increased in CUR5g-treated A549 cells (Fig. 1E). Following 24-h exposure to 10 μM CUR5g, numerous autophagosomes were observed in A549 cells, but much fewer in untreated controls (Fig. 1F). Based on these observations, CUR5g were selected for subsequent experiments.

### CUR5g causes a late-stage block of autophagy by suppressing the fusion of autophagosome with lysosome

**Fig. 2:**
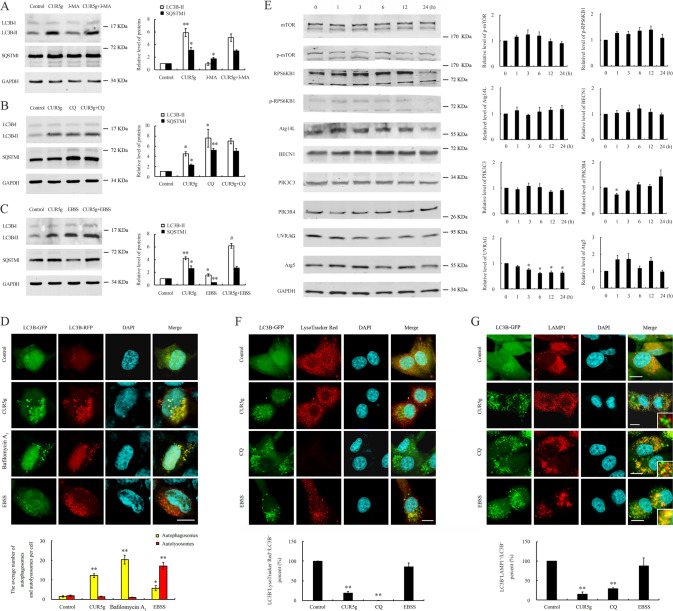
CUR5g inhibits autophagosome degradation by blocking autophagosome-lysosome fusion. **A** Western blot analysis of LC3B-II and SQSTM1 levels in A549 cells treated with DMSO or 10 μM CUR5g in the absence or presence of 3-MA (10 mM) for 24 h. GAPDH was used as a loading control. (*n* = 3; *p < 0.05, **p < 0.01 vs. Control). **B** Western blot analysis of LC3B-II and SQSTM1 levels in A549 cells treated with DMSO or 10 μM CUR5g in the absence or presence of CQ (30 μM) for 24 h. GAPDH was used as a loading control. (*n* = 3; *p < 0.05, **p < 0.01 vs. 0). **C** Western blot analysis of LC3B-II and SQSTM1 levels in A549 cells cultured in complete medium or EBSS in the absence or presence of CUR5g (10 μM) for 24 h. GAPDH was used as a loading control. (*n* = 3; *p < 0.05, **p < 0.01 vs. 0, ^#^*p* < 0.05 vs. CUR5g). **D** Representative fluorescence photographs of HEK293T cells stably expressing RFP-GFP-LC3B reporter. Cells were treated with DMSO or CUR5g (10 μM) in complete medium for 24 h, treated with EBSS for 6 h. Bafilomycin A_1_ (100 nM)-treated cells were used as positive controls. Nuclei were stained with DAPI. Scale bar = 10 μm. Histogram shows average number of autophagosomes (yellow) and autolysosomes (red) per cell. (*n* = 3; *p < 0.05; **p < 0.01 vs. control). **E** Western blot analysis of LC3B-II and SQSTM1 levels in A549 cells treated with 10 μM CUR5g for 0–24 h. GAPDH was used as a loading control. (*n* = 3; *p < 0.05 vs. 0). **F** Representative fluorescence photographs of the colocalization of GFP-LC3B and LysoTracker Red in U87 cells treated with DMSO, CUR5g (10 μM) for 24 h, treated with CQ (30 μM), or incubated with EBSS for 6 h. Nuclei were stained with DAPI. Scale bar = 10 μm. Histogram shows the percentage of LC3B^+^LysoTracker Red^+^ puncta (yellow) relative to LC3B^+^ puncta (green). (*n* = 3; **p < 0.01 vs. Control). **G** Representative fluorescence images of the colocalization of GFP-LC3B and LAMP1 in U87 cells treated with DMSO, CUR5g (10 μM) for 24 h, treated with CQ (30 μM), or incubated with EBSS for 6 h. Nuclei were stained with DAPI. Scale bar = 10 μm. Histogram shows the percentage of LAMP1^+^LC3B^+^ puncta (yellow) relative to LC3B^+^ puncta (green). (*n* = 3; **p < 0.01 vs. Control).

Exposure to 3-MA, the autophagosome formation inhibitor, did not eliminate CUR5g-induced SQSTM1 and LC3B-II accumulation (Fig. 2A). Treatment of A549 cells with CUR5g and the late-stage autophagy inhibitor CQ showed no effect on further increasing LC3B-II relative to simply CQ (Fig. 2B). CUR5g further induced LC3B-II accumulation within A549 cells under the nutrient deficient state compared with those cells maintained in nutrient adequate medium (Fig. 2C), indicating that CUR5g causes a late-stage block of autophagy.

Congruently, CUR5g increased the GFP^+^RFP^+^ punctum number (autophagosomes, yellow), but not GFP^-^RFP^+^ punctum number (autolysosomes, red), in HEK293T cells stably expressing RFP-GFP-LC3B reporter (Fig. 2D).

Additionally, CUR5g’s role in major proteins levels involved in autophagosome formation was analyzed, namely, the mechanistic target of rapamycin kinase (mTOR) and its substrate RPSK6B1, ATG14-like protein (Atg14L), BECN1, phosphatidylinositol 3-kinase catalytic subunit type 3 (PIK3C3), UV radiation resistance-associated gene (UVRAG), phosphoinositide 3-kinase regulatory subunit 4 (PIK3R4), as well as ATG5. Except for the significant reduction in UVRAG levels, the levels of other proteins in CUR5g-treated cells were not statistically different from control cells (Fig. 2E), further ruling out the possibility that CUR5g increases autophagy initiation.

The inhibition of autophagy can be induced by impaired autophagosome degradation or/and blocking of autophagosome-lysosome fusion. To clarify how CUR5g works, we detected the LC3B- LysoTracker Red colocalization within U87 cells. The overlap of LC3B-GFP signals with LysoTracker Red signals was observed in EBSS-incubated cells, rather than CUR5g-treated counterparts (Fig. 2F), indicating that CUR5g inhibits the fusion of autophagosomes and lysosomes. Notably, CUR5g did not reduce acid-dependent LysoTracker Red signals, unlike CQ, which accumulates within and alkalinizing the lysosome, suggesting that CUR5g does not block lysosome acidification. In parallel, LC3B puncta and lysosomal-associated membrane protein 1 (LAMP1, the lysosomal marker) were dramatically separated within CUR5G-treated cells, a phenomenon close to CQ-treated cells in a less pronounced manner (Fig. 2G), confirming that CUR5g blocks autophagosome-lysosome fusion.

### CUR5g does not affect the lysosomal proteolytic function and cytoskeleton, but blocks incorporation of syntaxin 17 (STX17) on autophagosomes

**Fig. 3:**
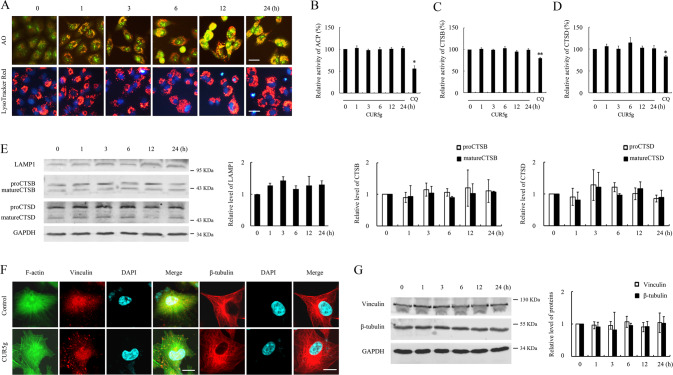
CUR5g does not impair the lysosomal proteolytic function and cytoskeleton. **A** Representative fluorescence photographs of acridine orange (AO) staining and LysoTracker Red staining of A549 cells treated with CUR5g (10 μM) for 0–24 h. Scale bar =10 μm. **B**–**D** Relative enzymatic activity of ACP (**B**), CTSB (**C**), and CTSD (**D**) in A549 cells treated with CUR5g (10 μM) for 0–24 h. CQ (30 μM)-treated cells were used as positive controls. (*n* = 3; *p < 0.05; **p < 0.01 vs. 0). **E** Western blot analysis of LAMP1, CTSB, and CTSD levels in A549 cells treated with 10 μM CUR5g for 0–24 h. GAPDH was used as a loading control. **F** Representative fluorescence photographs of the localization of F-actin (green), vinculin (red), and β-tubulin (red) in A549 cells treated with DMSO or CUR5g (10 μM) for 24 h. Nuclei were stained with DAPI. Scale bar = 10 μm. **G** Western blot analysis of vinculin and β-tubulin levels in A549 cells treated with 10 μM CUR5g for 0–24 h. GAPDH was used as a loading control.

We examined the effect of CUR5g on lysosomal pH with the use of Lysotracker red and acridine orange (AO) to further clarify whether CUR5g affects lysosomal function. We found that CUR5g did not attenuate red fluorescence in the above two dyes (Fig. 3A), indicating that CUR5g did not induce lysosomal alkalization. CUR5g did not suppress the activities of lysosomal proteases, including acid phosphatase (ACP), CTSB, and CTSD (Fig. 3B–D). In parallel, according to the WB assay, CUR5g had no effect on lysosomal marker LAMP1 expression, nor the conversions of proCTSB and proCTSD to mature cathepsins (Fig. 3E), supporting the notion that CUR5g does not disrupt lysosomal proteolytic function. Moreover, CUR5g treatment did not change the distribution of F-actin, vinculin, and β-tubulin (Fig. 3F). Vinculin and β-tubulin expression in CUR5g-treated cells were not significantly different from untreated cells (Fig. 3G), suggesting that CUR5g does not affect the cytoskeleton.

**Fig. 4:**
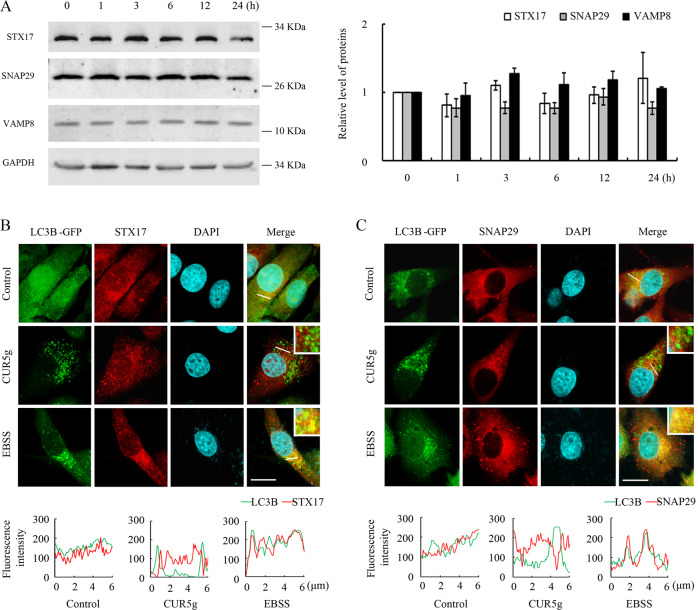
CUR5g blocks incorporation of STX17 on autophagosomes. **A** Western blot analysis of STX17, SNAP29, and VAMP8 levels in A549 cells treated with CUR5g (10 μM) for 0–24 h. GAPDH was used as a loading control. **B**, **C** Representative fluorescence images of the colocalization of LC3B (green) and STX17 (red) (**B**) or LC3B (green) and SNAP29 (red) (**C**). Nuclei were stained with DAPI. The line-scanned profiles show the distribution of fluorescence for each channel in the white line in the corresponding confocal images. Scale bar = 10 μm.

Autophagosomal membrane-localized STX17 interacts with endolysosome-localized vesicle-associated membrane protein 8 (VAMP8) and synaptosomal-associated protein of 29 kDa (SNAP29) to form a soluble N-ethylmaleimide-sensitive factor attachment protein receptor (SNARE) complex, and they play a key role in driving autophagosome-lysosome fusion [21]. As revealed by WB assay, CUR5g made no difference to reduce STX17, SNAP29, or VAMP8 expression (Fig. 4A). Immunostaining of cells treated with CUR5g showed that STX17 and SNAP29 were not recruited to LC3B-positive autophagosomes, whereas a significant colocalization of STX17 and SNAP29 with LC3B was observed in cells maintained in EBSS medium (Fig. 4B, C), suggesting that CUR5g prevented the incorporation of STX17 onto autophagosomes.

In addition to the SNARE complex, the role of Rab GTPase Rab7 in the fusion of autophagosomes with lysosomes has been extensively studied [22, 23]. We found that CUR5g did not influence Rab7 level (Fig. S4A). Transfection of A549 cells with wild-type pEGFP-Rab7 or the mutants Q67L (a constitutively active form) and T22N (a dominant negative form) did not reverse CUR5g-induced accumulation of LC3B-II and SQSTM1 (Fig. S4B), ruling out the possibility that CUR5g suppressed autophagosome-lysosome fusion in a Rab7-dependent manner.

### Overexpression of UVRAG reverses CUR5g-induced autophagosome accumulation

**Fig. 5:**
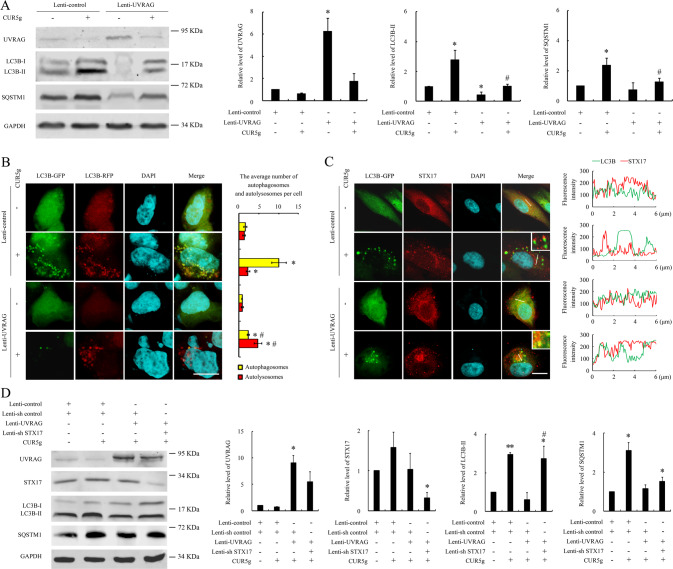
Overexpression of UVRAG reversed the inhibitory effect of CUR5g on autophagy, which can be eliminated by knocking down STX17. **A** Western blot analysis of UVRAG, LC3B-II, and SQSTM1 levels in A549 cells transfected with control lentivirus or UVRAG lentivirus in the presence of DMSO or CUR5g (10 μM) for 24 h. GAPDH was used as a loading control. (*n* = 3; *p < 0.05 vs. Lenti-control, ^#^*p* < 0.05 vs. Lenti-control + CUR5g). **B** Representative fluorescence photographs of HEK293T cells stably expressing RFP-GFP-LC3B reporter. Cells were transfected with control lentivirus or UVRAG lentivirus in the presence of DMSO or CUR5g (10 μM) for 24 h. Nuclei were stained with DAPI. Scale bar = 10 μm. Histogram shows average number of autophagosomes (yellow) and autolysosomes (red) per cell. (*n* = 3; *p < 0.05 vs. Lenti-control, ^#^*p* < 0.05 vs. Lenti-control + CUR5g). **C** Representative fluorescence images of the colocalization of LC3B (green) and STX17 (red). Cells were transfected with control lentivirus or UVRAG lentivirus in the presence of DMSO or CUR5g (10 μM) for 24 h. Nuclei were stained with DAPI. The line-scanned profiles at the right of each confocal image show the distribution of fluorescence for each channel in the white line in the corresponding confocal images. Scale bar = 10 μm. **D** Western blot analysis of UVRAG, STX17, LC3B-II, and SQSTM1 levels in A549 cells transfected with control lentivirus, control shRNA lentivirus, UVRAG lentivirus or/and STX17 shRNA lentivirus in the presence of DMSO or CUR5g (10 μM) for 24 h. GAPDH was used as a loading control. (*n* = 3; *p < 0.05, *p < 0.01 vs. Lenti-control + Lenti-sh control, ^#^*p* < 0.05 vs. Lenti-sh control + Lenti-UVRAG + CUR5g).

In addition to being a key component in autophagosome formation, UVRAG is also thought to be necessary for autophagosome-lysosome fusion [24]. The fact that UVRAG levels remarkably decreased within CUR5g-exposed cells (Figs. 2E and S5), making us investigate whether the failure of autophagosome-lysosome fusion is due to UVRAG downregulation. We found that in UVRAG overexpressing cells, CUR5g failed to induce the accumulation LC3B-II and SQSTM1, increase the number of GFP^+^RFP^+^ puncta, or prevent the recruitment of STX17 to autophagosomes (Fig. 5A–C). Overexpression of UVRAG did not prevent CUR5g-induced SQSTM1 and LC3B-II accumulation within STX17 knockdown cells (Fig. 5D). These results collectively demonstrated that CUR5g inhibits STX17 targeting to autophagosomes by down-regulating UVRAG.

The molecular modeling and docking studies predicted that CUR5g appears to bind preferably to the region 270–442 of UVRAG (Fig. S6) which was sufficient for interaction with VPS16 [25]. Efficient binding to VPS16 is necessary for UVRAG to fully facilitate autophagosome-endosome/lysosome fusion [25], raising the possibility that CUR5g might weaken the interaction between UVRAG and VPS16 and therefore impairs the maturation of the autophagosome. Supporting this notion, we found that CUR5g weakened UVRAG-VPS16 interaction by immunofluorescence observation and coimmunoprecipitation analysis (Fig. S7).

### CUR5g exerts anticancer effects on A549 cells

**Fig. 6:**
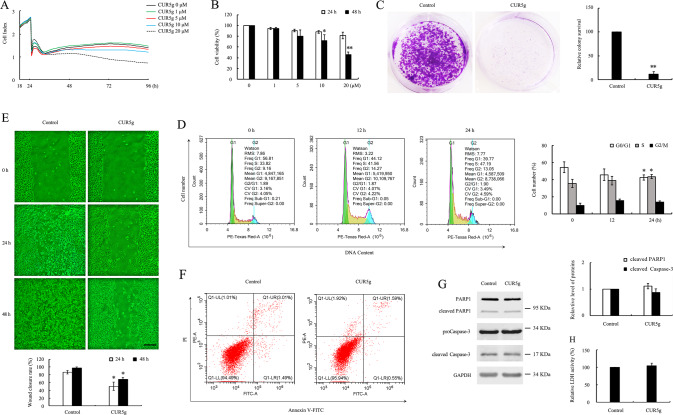
CUR5g inhibited proliferation and migration of A549 cells, but did not induce apoptosis or necrosis. **A** Cell number was monitored over 96 h in a real-time manner using an xCELLigence RTCA S16 System. Cell-sensor impedance is displayed as the cell index. CUR5g (0–20 μM) was added after the cells were seeded into the plates for 24 h. **B** MTT assays. **C** Representative images of colony formation assay of A549 cells treated with DMSO or CUR5g (10 μM) for 12 days. At the end, the colonies were fixed and stained with crystal violet. Histogram shows the relative colony survival. (*n* = 3; **p < 0.01 vs. Control). **D** Flow cytometric analysis of cell cycle in A549 cells treated with CUR5g (10 μM) for 0, 12, or 24 h, respectively. **E** The effect of DMSO or CUR5g (10 μM) on the migratory potential of A549 cells was analyzed through a wound-healing assay. Microscopy photographs were taken after treatment for 0, 24, or 48 h, respectively. Scale bar = 100 μm. Histogram showed the wound closure rate. (*n* = 3; *p < 0.05 vs. control). **F** Flow cytometric analysis of Annexin V-FITC/PI staining in A549 cells treated with DMSO or CUR5g (10 μM) for 24 h. **G** Western blot analysis of cleaved PARP1 and cleaved Caspase-3 in A549 cells treated with DMSO or CUR5g (10 μM) for 24 h. GAPDH was used as a loading control. **H** Bar graph shows the relative LDH activity in A549 cells treated with DMSO or CUR5g (10 μM) for 24 h.

A549 cell number slightly decreased after treatment with 10 µM CUR5g, while CUR5g at 20 µM decreased the number of A549 cells significantly (Fig. 6A), suggesting that CUR5g exhibited great toxicity to A549 cells at 20 μM. Similar results were obtained by MTT assays (Fig. 6B). However, CUR5g at 40 µM showed no discernable activity in healthy human umbilical vein endothelial cell (HUVEC) viability (Fig. S8). We found that UVRAG was significantly higher in both mRNA and protein levels in HUVECs than in A549 cells (Fig. S9A, B). More excitingly, at the concentration below or equal to 50 μM, CUR5g did not induce a statistically significant reduction in UVRAG levels (Fig. S9C), and the interaction between UVRAG and VPS16 is not significantly affected by CUR5g (Fig. S9D), suggesting that A549 cells might be more sensitive to CUR5g because they exhibit lower basal levels of UVRAG. These results at least partially explained why CUR5g showed more selective autophagy inhibitory effects and cytotoxic activity against NSCLC cells than normal HUVECs. In addition, 10 µM CUR5g almost completely suppressed the colony formation in A549 cells (Fig. 6C). Upon CUR5g treatment, G0/G1-phase cell proportion declined, whereas S phase proportion elevated (Fig. 6D), suggesting that CUR5g promoted cell cycle arrest of A549 cells at S phase.

CUR5g at 10 µM almost completely inhibited colony formation, promoting us to examine whether CUR5g affects the mobility of A549 cells. As revealed by a scratch assay, CUR5g significantly inhibited A549 cell migration (Fig. 6E). After treatment with CUR5g, Annexin V-positive cell proportion (Fig. 6F) and levels of cleaved PARP1 or cleaved Caspase-3 did not increase (Fig. 6G), nor did the activity of lactic acid dehydrogenase (Fig. 6H), suggesting that CUR5g did not induce apoptosis and necrosis of A549 cells. Similarly, CUR5g made no difference to intracellular and mitochondrial ROS, nor the MMP levels (Fig. S10 and S11).

### CUR5g exhibits potent synergistic anticancer effects with cisplatin on A549 cells and inhibits autophagic flux in vivo

**Fig. 7:**
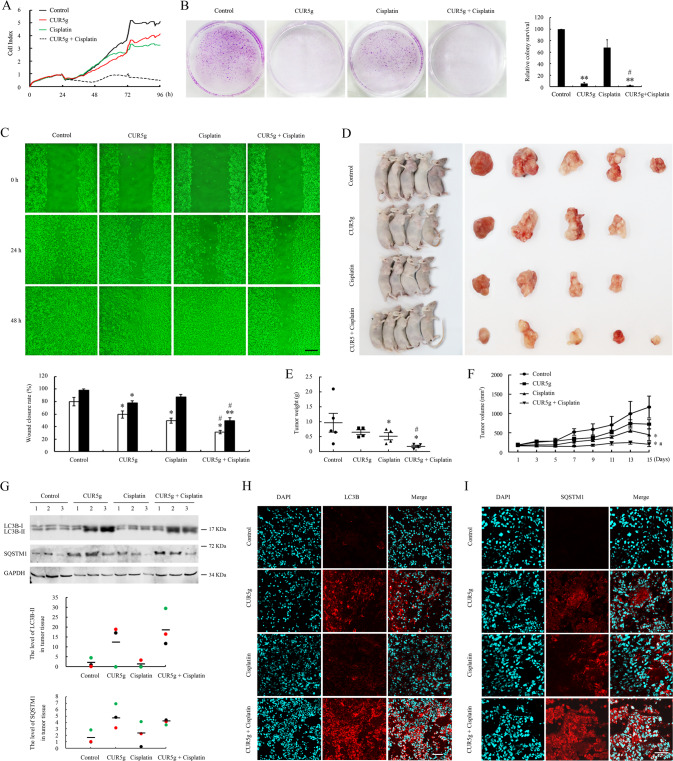
CUR5g exhibits synergistic anticancer effects with cisplatin and inhibits autophagic flux in vivo. **A** Cell number was monitored over 96 h in a real-time manner using an xCELLigence RTCA S16 System. DMSO, CUR5g (10 μM), cisplatin (30 μM), or CUR5g (10 μM) plus cisplatin (30 μM) was added after the cells were seeded into the plates for 24 h. **B** Representative images of colony formation assay of A549 cells treated with DMSO, CUR5g (10 μM), cisplatin (30 μM), or CUR5g (10 μM) plus cisplatin (30 μM) for 12 days. Histogram shows the relative colony survival. (*n* = 3; **p < 0.01 vs. Control, ^#^*p* < 0.05 vs. cisplatin). **C** The effect of DMSO, CUR5g (10 μM), cisplatin (30 μM), or CUR5g (10 μM) plus cisplatin (30 μM) on the migratory potential of A549 cells was analyzed through a wound-healing assay. Microscopy photographs were taken after treatment for 0, 24, or 48 h, respectively. Scale bar = 100 μm. Histogram showed the wound closure rate. (*n* = 3; *p < 0.05; **p < 0.01 vs. control, ^#^*p* < 0.05 vs. cisplatin). **D** Images show all the animals and tumors in the experiments. **E** Tumor weight was measured on the day of sacrifice. (Each group mice n = 4–5; *p < 0.05 vs. control, ^#^*p* < 0.05 vs. cisplatin). **F** Tumor volume were recorded every 2 days for up to 15 days. (Each group mice *n* = 4–5; *p < 0.05 vs. control, ^#^*p* < 0.05 vs. cisplatin). **G** Western blot analysis of LC3B-II and SQSTM1 in tumor tissues. 3 tumor tissues were randomly selected from each group for analysis. GAPDH was used as a loading control. Scattergram shows the densitometric analysis of LC3B-II and SQSTM1 in tumor tissues. **H**, **I** Representative fluorescence images of LC3B (**H**) or SQSTM1 (**I**) in tumor sections. Nuclei were stained with DAPI. Scale bar = 80 μm.

We further tested whether CUR5g could synergize with cisplatin to exhibit stronger anticancer activity against NSCLC. Different from the cells exposed to CUR5g or cisplatin alone, the number of A549 cells co-treated with CUR5g and cisplatin displayed a rapid decrease, and this trend maintained until the end of the experiment (Fig. 7A). Likewise, data from colony formation assay and wound-healing assay showed that CUR5g enhanced cisplatin’s anticancer activity in A549 cells (Fig. 7B, C). Moreover, we compared the effectiveness of CUR5g with other three autophagy regulators including curcumin, CQ, and CA-5f in combination with cisplatin. At the same concentration of 10 µM, CUR5g/cisplatin combination induced more effective synergistic cytotoxicity to A549 cells than the combination of cisplatin with curcumin or CA-5f. The effectiveness of 10 µM CUR5g combined with cisplatin is similar to 30 µM CQ combined with cisplatin (Fig. S12).

To explore whether the combination of CUR5g and cisplatin serves a similar function in vivo, a xenograft nude mouse model of NSCLC was established by subcutaneously injecting A549 cells. CUR5g alone or combined with cisplatin showed well tolerance in nude mice, and abnormal physical signs were not reported in the whole procedure. Differences in average body weight (BW) among all treatments were not significant (Fig. S13). No signs of mortality or macroscopic abnormalities in the organs were discovered, and no obvious pathological alterations were observed in the major organs (Fig. S14). The size and weight of the dissected tumors in the CUR5g and cisplatin combination group remarkably decreased compared with the remaining groups (Fig. 7D, E). As expected, CUR5g or cisplatin alone retarded the growth of xenografted tumors, whereas the combination treatment almost completely inhibited tumor growth (Fig. 7F), demonstrating that CUR5g potently enhanced cisplatin’s anticancer activity in A549 cells in vitro as well as in vivo. Consistent with in vitro data, as revealed by WB assay, CUR5g increased LC3B-II and SQSTM1 levels within tumor tissues (Fig. 7G). The level of LC3B-II in CUR5g plus cisplatin group significantly increased relative to cisplatin group (Fig. 7G). Immunofluorescence staining showed a significant increase of punctate LC3B and SQSTM1 signals in tumor tissues obtained from CUR5g-treated group as relative to control (Fig. 7H, I). CUR5g synergistically promoted the effect of cisplatin on LC3B accumulation in tumors (Fig. 7H, I). In conclusion, the above findings suggested that CUR5g promoted the cisplatin sensitivity of A549 cells by inhibiting autophagic flux.

## Discussion

Countless evidence has demonstrated that autophagy helps NSCLC to resist various chemotherapeutic drugs, and thus has emerged as an attractive target to overcome drug resistance [16, 17, 26]. A growing number of clinical trials have been proposed or are undergoing to evaluate the anticancer effect of autophagy inhibitors combined with chemotherapeutic drugs (http://clinicaltrials.gov). However, the vast majority of autophagy inhibitors identified so far, especially CQ and its derivative HCQ, actually disrupt lysosomal and/or possible non-lysosomal events different from autophagy [27]. In fact, the anticancer therapeutic effect of HCQ and CQ may be achieved through modulating autophagy-independent machinery, as evidence by the unsatisfactory inhibitory effect of high-dose HCQ on autophagy in clinical trials [11, 27]. Thus, the discovery of more selective and specific autophagy inhibitors is a prerequisite for preclinical validation studies.

This work discovered that CUR5g was the new autophagy inhibitor that can affect autophagy within diverse cancer cell types, but not within non-tumor cells. CUR5g inhibits late autophagy by suppressing autophagosome-lysosome fusion. A wide majority of late-stage autophagy inhibitors are actually lysosomal inhibitors, which alkalinize intralysosomal pH or/and impair the lysosomal function, thereby resulting defect in lysosomal degradation of autophagosomes. One of the limitations of these lysosome inhibitors is the lack of specificity to target autophagy, which may affect other metabolic pathways and cause lysosomal storage disease [28, 29]. CUR5g did not influence lysosomal acidification and hydrolytic function, nor did it affect lysosome quantity, indicating that this small-molecule compound is a non-lysosomal targeting inhibitor.

The trafficking of autophagosomes and their fusion with lysosomes are mainly depend on cytoskeletal systems including actin and microtubules [30]. CUR5g had no significant effect on cytoskeleton of A549 cells, as indicated by unchanged reticular or fascicular distribution of F-actin and β-tubulin, and vinculin and β-tubulin expression was not significantly different. Assembly of the SNARE complex is another critical step in membrane fusion. During autophagy, STX17 will be recruited into the complete autophagosomes, followed by interaction with SNAP29 as well as endosomal/lysosomal VAMP8 for forming the SNARE complex, thus providing the energy necessary in autophagosome-lysosome fusion [21]. CUR5g impeded STX17 translocation onto autophagosomes by down-regulating UVRAG, thereby rendering autophagosomes “fusion failure.” A previous study indicated that UVRAG not only regulates the formation of autophagosomes but also facilitates their fusion with lysosomes [25]. However, later studies showed that UVRAG is mainly involved in lysosomal degradation, while it is dispensable for autophagosome-lysosome fusion, and thus it may regulate autophagy indirectly [24, 31]. Although the precise role of UVRAG in autophagosome-lysosome fusion is controversial, our results showed that CUR5g-induced UVRAG reduction is the main reason that hinders STX17 loading onto autophagosomes. As mentioned above, routinely-used late autophagy inhibitors like CQ and bafilomycin A1 may not be the ideal options as they are actually lysosomal inhibitors, which impede all lysosomal degradation pathways. Even the commonly used early-stage autophagy inhibitors such as 3-MA and wortmannin are non-specific as they suppress all phosphatidylinositol 3-kinase-associated pathways, leading to a plethora of side effects. Our results showed that CUR5g interfered with the recruitment of STX17 onto autophagosomes thereby hindering the maturation of autophagosomes. Blockade of STX17 is more specific than use of lysosomal inhibitors in inhibiting autophagic flux [32], such observations support the idea that using CUR5g, a small-molecule compound blocks the incorporation of STX17 onto autophagosomes, might represent a cleaner way to inhibit autophagosome degradation.

More excitingly, CUR5g selectively induced autophagosome accumulation within tumor cells, rather than healthy cells, which might avoid undesirable side effects. Supporting this notion, CUR5g showed no obvious toxicity to normal HUVECs in *vitro* or mice in *vivo*. CUR5g alone did not induce apoptosis and necrosis of A549 cells, but significantly inhibited the mobility and colony formation of A549 cells. In animals, the anticancer effect of CUR5g was limited, while its combination with cisplatin almost completely inhibited lung adenocarcinoma growth without induction of the body weight loss and histological changes of vital organs. In A549 xenografts, CUR5g administration markedly promoted LC3B-II and SQSTM1 levels by immunofluorescence staining and western blot analysis, confirming the efficiency of CUR5g in inhibiting autophagy of lung adenocarcinoma in *vivo*. However, whether there is a link between CUR5g-induced S phase arrest and autophagy inhibition remains unknown. Previous studies provided some insight into this concern. Silencing of autophagy-related ATG5 arrested cells in S phase [33], a phenotype similar to the cells that were treated with autophagy inhibitors CQ [34], suggesting a possibility that CUR5g-induced autophagy inhibition might contribute to S phase arrest in A549 cells. It has been reported that cisplatin facilitated cell cycle arrest in lung cancer cells [35, 36]. A further S phase arrest induced by CUR5g may make the damage exceed the threshold that cells can bear, which may be another potential reason for the synergy of cisplatin and CUR5g. A combination treatment of the autophagy inhibitor 3-MA and cisplatin elevated S phase cell proportion as well as apoptosis rate [37]. Such observations support this possibility. Whether and how S phase arrest is associated with CUR5g-induced autophagy inhibition and synergistic effect with cisplatin should be further explored.

In summary, this work identifies CUR5g as the specific late autophagy inhibitor without affecting other cellular processes (lysosomal acidification and hydrolysis function, cytoskeleton, Rab7, and ROS). Our study provides a novel tool for selectively inhibiting autophagy in cancer cells and is a potential candidate to develop for the treatment of NSCLC in conjunction with cisplatin. We will explore the direct targets of CUR5g and examine the anticancer effect of CUR5g combined with other first-line chemotherapy regimens in our future study.

## Methods

### Materials

A series of curcumin analogs, including CUR5g (>99% purity as determined through high-performance liquid chromatography), were previously synthesized at our lab. All tested analogs were subject to dissolution within dimethyl sulfoxide (DMSO) for preparing the stock solution (10 mM) diluted in different doses prior to application. This work obtained fetal bovine serum (FBS; SH30084.03) in HyClone (Logan, Utah, USA), RPMI 1640 medium (11875101), and DMEM medium (11995065) in Gibco (Grand Island, NY, USA). Trypsin (A003702), DMSO (A100231-0500), bis-acrylamide (A600025-0250), tris (A600194-0500), ammonium persulfate (A600072-0100) and sodium dodecyl sulfate (SDS; A100227) were provided by Shanghai Sangon Biotech (Shanghai, China). Protease inhibitor cocktail (P8340), Earle’s balanced salt solution (EBSS; E2888), chloroquine (CQ; C6628), 3-methyladenine (3-MA; M9281), acridine orange (AO; 235474), thiazolyl blue tetrazolium bromide (MTT; M2128), curcumin (C1386) and 4,6-diamidino-2-phenylindole dihydrochloride (DAPI, D8417) were provided by Sigma-Aldrich (St. Louis, MT, USA). MitoSOX™ red mitochondrial superoxide indicator (M36008), Premo™ autophagy sensor LC3B-GFP (BacMam 2.0; P36235), Lipofectamine™ 2000 CD Transfection Reagent (12566014) and Lysotracker Red DND (L7528) were provided by Invitrogen (Carlsbad, CA, USA). This work acquired YF dye phalloidin conjugates in US Everbright, Inc. (Suzhou, China), whereas FITC Annexin V Apoptosis Detection Kit I (556547) in BD Biosciences (NJ, USA). Meanwhile, the present study acquired the primary antibody against PARP1 (AP102), Lyso-Tracker Red (C1046), JC-1 probe (C2006), Ad-GFP-LC3B (C3006), Reactive Oxygen Species Assay Kit (S0033), ACP assay kit (P0326), apoptosis inducers kit (C0005), PI (C1052), BeyoMag Anti-Flag Magnetic Beads (P2115) and polybrene (C03511) in Beyotime Biotechnology (Shanghai, China). GoldView I nucleic acid dye (G8140) and bovine serum albumin (BSA, A8020) were provided by Solarbio (Beijing, China). Meanwhile, this work acquired Cathepsin B (K140) and D (K143) activity fluorometric assay kits in BioVision (CA, USA). This work acquired lactate dehydrogenase assay kit (A020-1) in NanJing JianCheng (Nanjing, China), whereas RaPure total RNA kit (R4011-02) in Magen (Guangzhou, China), whereas 2× Taq Master Mix (Dye Plus; P112), AceQ® qPCR SYBR® Green Master Mix (Q111), and HiScript®II Q RT SuperMix for qPCR (R223-01) in Vazyme Biotechnology (Beijing, China). 4% paraformaldehyde (MA0192) was purchased from meilunbio (Dalian, China). pHAGE-CMV-MCS-IRES-ZsGreen1 vectors (P4867) were obtained from miaolingbio (Wuhan, China). pLKO.1 vectors (VT1792) were obtained from YouBio (Guangzhou, China). PMD2.G (12259) and PsPAX2 (12260) were purchased from addgene (Cambridge, MA, USA). Crystal violet (1199) was provided by DAMAO CHEMICAL REAGENT FACTORY (Tianjin, China). In the meantime, this work purchased Hematoxylin and Eosin Staining Kit (G1005) in Servicebio (Wuhan, China). Anti-GAPDH (G9545), anti-SQSTM1 (P0067), anti-LC3B (L7543), anti-PIK3R4 (HPA036032), anti-CTSB (SAB1405676), anti-CTSD (SAB2106553), anti-mTOR (T2949), anti-p-mTOR (SAB4504476), anti-PIK3C3 (V9764), and anti-UVRAG (SAB4200005) primary antibodies were provided by Sigma-Aldrich (St. Louis, Montana, USA). Anti-RPS6KB1 (34475 S), anti-phospho-RPS6KB1 (9206), anti-Atg14L (96752), anti-LAMP1 (15665) and anti-ATG5 (12994) primary antibodies were provided by Cell Signaling Technology (MA, USA). Anti-vinculin (26520-1-AP) and anti-VPS16 (17776-1-AP) primary antibodies were provided by Wuhan Sanying Biotech (Wuhan, China). Anti-β-tubulin (A01030) primary antibody was provided by Abbkine (California, USA). Anti-Beclin 1 (AP6020) primary antibody was provided by Bioworld (MN, USA). Anti-STX17 (ab245637), anti-VAMP8 (ab89158) and anti-SNAP29 (ab181151) primary antibodies, and anti-Alexa Flour® 647 goat anti-rabbit IgG secondary antibody (150079) were provided by Abcam (Cambridge, MA, USA). Anti-IRDye® 680RD goat anti-mouse (926-68070), anti-IRDye® 680RD goat anti-rabbit (926-68071), anti-IRDye® 800CW goat anti-mouse (925-32210) and anti-IRDye® 800CW goat anti-rabbit (925-32211) secondary antibodies were provided by Li-Cor Biosciences (NE, USA). Anti-Alexa Flour® 488 goat anti-mouse IgG (ZF-0512) and anti-Alexa Flour® 488 goat anti-rabbit IgG (ZF-0511) secondary antibodies were provided by Beijing ZSGB-BIO (Beijing, China). Cisplatin (HY-17394) and bafilomycin A_1_ were purchased from MedChemExpress (NJ, USA). The plasmid Rab7-Q67L and Rab7-T22N were kindly provided by Dr. Youli Jian (State Key Laboratory of Molecular and Developmental Biology, Institute of Genetics and Developmental Biology, Chinese Academy of Sciences, Beijing, China).

#### Cell culture

This work obtained A549 (SCSP-503) and MCF-7 (SCSP-531) cell lines in Cell Bank of Chinese Academy of Sciences (http://www.cellbank.org.cn), whereas Adriamycin-resistant MCF-7 breast cancer (BC) cells (MCF-7/ADR, CL-0522) in Procell (Wuhan, China), and H157 (CRL-5802TM) and HepG2 (HB-8065™) cells in American Type Culture Collection. In addition, this work acquired BEAS-2B (GDC139) cells in China Center for Type Culture Collection (http://www.cctcc.org). DH5α competent cells (DL1001) were purchased from Shanghai Weidi Biotechnology. U87 and HEK293T were kindly provided by Dr. Jing Liu (Laboratory of Microvascular Medicine, Medical Research Center, the First Affiliated Hospital of Shandong First Medical University, Jinan, China). HBMECs were kindly provided by Dr. Haiying Li (Department of Neurosurgery & Brain and Nerve Research Laboratory, the First Affiliated Hospital of Soochow University, Suzhou, China.). This work acquired HUVECs at our lab according to the previous description [38] and cultivated them within MCDB-131 medium (Sigma-Aldrich, M8537) that contained 70 ng/ml fibroblast growth factor 2 (FGF-2, GIBCO, 13256-029) as well as 20% heat-inactivated FBS. In addition, this work kept A549 and H157 cell lines within RPMI 1640 medium, whereas U87, HEK293T, HepG2, MCF-7, and MCF-7/ADR cell lines within DMEM, and BEAS-2B cell line within a base medium (China Center for Type Culture Collection). These cells were all cultivated within the medium that contained 1% penicillin/streptomycin (PS) (Solarbio, P1400) and 10% FBS and incubated under 37 °C and 5% CO_2_ conditions.

#### Western blot (WB) assay

Following proper treatments, both cells and tissues were subject to lysis within lysis buffer that contained 2% SDS, 25 mM Tris-HCl (pH 6.8), 2 mM PMSF (Sigma-Aldrich, P7626), 6% glycerol, 0.02% bromophenol blue, 1% 2-mercaptoethanol (Sigma-Aldrich, 07604), as well as the protease inhibitor cocktail. The lysates were boiled for 15 min and immunoblotting were performed according to standard methods. After incubation of membrane using IRDye 680 or IRDye 800 secondary antibody, the Li-Cor Odyssey infrared imager (Li-Cor Biosciences, Lincoln, NE) was utilized for band scanning. For quantification, Odyssey Application Software was used to determine the band intensity.

#### Quantitative real-time PCR (qRT-PCR)

This work collected total RNA and prepared it into cDNA according to specific protocols. The LC3B and SQSTM1 mRNA expression was analyzed by 2^−ΔΔCt^ approach, with GAPDH being a normalization reference. The corresponding primers: LC3B-F: 5’-AAACGCATTTGCCATCACAGT-3’, LC3B-R: 5’-GTGAGGACTTTGGGTGTGGTTC-3’; SQSTM1-F: 5’-TACGACTTGTGTAGCGTCTGC-3’, SQSTM1-R: 5’-GTGTCCGTGTTTCACCTTCC-3’; GAPDH-F: 5’-AATGACCCCTTCATTGAC-3’, GAPDH-R: 5’-TCCACGACGTACTCAGCGC-3’.

#### Transmission electron microscopy

After growing of A549 cells till about 80% density within the 100-mm dishes, cells were subject to 24-h DMSO or CUR5g (10 µM) treatment, followed by fixation using 2.5% glutaraldehyde, dehydration using the graded ethanol series before being embedded in epoxy resin and sectioning. Cells were then stained by aqueous uranyl acetate and later by lead citrate, finally, the JEM-1230 transmission electron microscopy (JEOL Co., Ltd., Japan) was utilized for photographing of ultrathin sections.

#### Immunofluorescence (IF) analysis and confocal microscopy

After growing of U87 cells till around 80% confluency within the 14-mm round-glass coverslips, cells were transfected with Ad-GFP-LC3B, followed by 24-h treatment using DMSO, CUR5g (10 µM) or 6-h incubation with CQ (30 μM) or EBSS. Afterwards, cells were subject to fixation using 4% paraformaldehyde (PFA), blocking with 5% BSA within PBS that contained Tween-20 (PBST), labeling using corresponding primary antibodies under 4 °C overnight, and 30-min incubation using secondary antibodies under 37 °C. DAPI was added for nuclear counter-staining. Finally, the cells were photographed with the confocal laser-scanning microscope (Olympus FLUOVIEW FV3000). Fluorescence distribution between white lines in the corresponding images was conducted by ImageJ software (National Institutes of Health).

#### AO, LysoTracker Red, and F-actin staining

After growing A549 cells till around 80% confluency within 24-well plates, cells were exposed to DMSO or CUR5g (10 µM) at various times, followed by 10-min staining using 5 μg/mL AO under 37 °C or 15-min staining using 1 mmol/L Lysotracker Red under 37 °C, respectively. Following the manufacturer’s instructions, after 15-min fixing in 4% PFA, cell staining with phalloidin conjugates (200 μL) was conducted for detecting F-actin. Nuclei were counterstained with DAPI.

#### Detection of ACP, CTSB, and CTSD enzymatic activities

Commercially available assay kits were used to detect ACP, CTSB, and CTSD enzymatic activities in line with specific instructions. The ACP activities were measured as the absorbance with the Epoch TM spectrophotometer (BioTek Instruments) at 405 nm. For CTSB, fluorescence was detected at the emission and excitation wavelengths of 505 and 400 nm, separately, whereas 460 and 328 nm separately for CTSD using a microplate reader (PerkinElmer EnSpire).

#### Construction and identification of overexpression or shRNA lentiviral vectors

The sequences used for UVRAG overexpression were shown below: F-5’-GCGACGCGTGCCACCATGAGCGCCTCCGCGTCGGT-3’, R-5’ CCGCTCGAGCTTATCGGAACTCCTGCGCGGCCG-3’. Each sequence was cloned in Mlu I- and Xhol I-cleaved pHAGE-CMV-MCS-IRES-ZsGreen1 vectors. The shRNA sequences targeting STX17 were shown below: F-5’-CCGGCGATCCAATATCCGAGAAATTCTCGAGAATTTCTCGGATATTGGATCGTTTTTG-3’, R-5’-AATTCAAAAACGATCCAATATCCGAGAAATTCTCGAGAATTTCTCGGATATTGGATCG-3’. Each sequence was cloned in Age I- and EcoR I-cleaved pLKO.1 vectors. Calcium chloride was utilized to prepare DH5α competent cells for subsequent transfection. PCR assay was conducted to select positive clones, which were sent to Sangon Biotech for sequencing.

#### Lentivirus collection and transfection

Target plasmid (1 µg) was co-transfected with 250 ng PMD2.G and 750 ng PsPAX2 into HEK293T cells. At 48 h later, medium containing lentivirus was harvested for 30-min centrifugation at 6000 rpm to collect supernatants, which were then filtered using the 0.45-µM filter (210910, BIOFIL), followed by 2-h centrifugation at 20,000 rpm and 4 °C. After growing of A549 cells till about 60% confluency within the 6-well plates, cells were subject to infection using lentivirus suspension that contained 4 µg/ml polybrene. At 4 h later, polybrene was diluted with freshly prepared medium (1 ml). At 48-h post-transfection, fresh medium that contained 4 μg/mL purinomycin was added to incubate cells for another 24 h. Overexpression or knockdown efficiency was identified by PR-PCR and WB assays.

#### Cell viability assay

This work inoculated A549 cells in E-Plate 16-well plates, followed by treatment using DMSO, a series of concentrations of CUR5g (1–20 µM), cisplatin (30 µM), CUR5g (10 µM) and cisplatin (30 µM). The cells were continuously monitored for 96 h with xCELLigence RTCA S16 System (ACEA Biosciences). The changes in cell-electrode impedance were converted to cell index representing cell viability with RTCA S16 Software (version 1.0). In addition, MTT assay was carried out to analyze cell viability. After inoculation of cells into the 96-well plates, they were subject to treatment using DMSO or a series of concentrations of CUR5g (1–20 µM). After discarding the original medium, freshly prepared medium (100 μL) containing MTT solution was introduced into all wells, followed by 4-h incubation. The microplate reader (Tecan Spark, Switzerland) was employed for measuring cell viability at 490 nm.

#### Colony formation assay

After inoculation of A549 cells (3 × 10^3^) in six-well plates, DMSO, CUR5g (10 µM), cisplatin (30 µM), CUR5g (10 µM), and cisplatin (30 µM) were added every 2 days for 12 days, followed by 4% PFA fixation and 0.5% crystal violet staining for colony visualization.

#### Cell cycle analysis

After growing to ~60% density within the 100-mm dishes, A549 cells were treated with 12/24-h DMSO or CUR5g (10 µM), respectively. After treatment, they were collected and immersed in cold 70% ethanol for 4 h, followed by centrifuging at 1000 g for 5 min and staining with PI for a 30-min period under 37 °C. At last, flow cytometry (FCM, BD FACSCalibur, Franklin Lakes, NJ, USA) was performed for cell analysis.

#### Scratch assay

After growing of A549 cells till density within the 24-well plates, scratches were made in the well center using the sterile pipette tip. Cells were later washed and treated by DMSO, CUR5g (10 µM), cisplatin (30 µM), CUR5g (10 µM), and cisplatin (30 µM). Images were made immediately, 24 h or 48 h after scratching. Data were analyzed using ImageJ software.

#### Annexin V-PI double-staining

After growing till about 80% density within the 100-mm dishes, A549 cells were treated with 24-h DMSO or CUR5g (10 µM), followed by cell collection and staining using FITC apoptosis detection kit I in line with specific instructions. Data acquisition and analysis using flow cytometry.

#### Animal experiments

All in vivo experimental protocols were carried out following Guide for the Care and Use of Laboratory Animals released by US National Institutes of Health (NIH Publication No. 85-23, revised 1996). Each experiment gained approval from Animal Care Committee of Laboratory Animal Center of Zhengzhou University. (Approval no. ZZU-LAC20211015 [17]).

This study obtained BALB/c nude mice (4-week-old) in Beijing Vital River Laboratory Animal Technology Co., Lt and maintained them within the specific pathogen-free (SPF) environment at Laboratory Animal Center from School of Medical Sciences of Zhengzhou University (Henan Province, China). 1 × 10^6^ A549 cells were subcutaneously injected into the right scapula of each nude mouse. At 14-day post-implantation of A549 cells, palpable tumors were observed in each mouse. Later, mice were randomized into 4 groups (*n* = 4 or 5 each group). CUR5g (40 mg/kg), cisplatin (1 mg/kg), CUR5g (40 mg/kg) and cisplatin (1 mg/kg) were injected via caudal vein every 2 days for up to 15 days. Control mice were given injection of the same concentration of DMSO. A micrometer was employed for measuring tumor size at 2-day intervals from the initial injection. This work determined tumor volume by tumor length × (square of width)/2. All animals were sacrificed after the last injection for 48 h, and the tumors and major organs were removed and weighed immediately. Each major organ was subject to embedding in optimal cutting temperature (OCT) compound (Tissue-Tek), followed by slicing in the 7-μm slices using the ultrathin semiautomatic microtome (CM1850, Leica, Wetzlar, Germany). For histologic examination, cryosections were subject to H&E staining.

#### Statistical analysis

Each experiment was carried out twice in triplicate. Average values between two groups were compared by unpaired Student’s *t* test, while multiple groups were compared by ANOVA. Statistical significance was assigned when *p < 0.05 or **p < 0.01 with SPSS17.0. (Almonk, New York, USA). Results were represented by mean ±SD.

## Supplementary information

Full length WB Original Data

Supplementary information-re

## Data Availability

The datasets used and analyzed during the current study are available from the corresponding author upon reasonable request.
