# Supplementary material for: CUR5g, a novel autophagy inhibitor, exhibits potent synergistic anticancer effects with cisplatin against non-small-cell lung cancer
Source: Cell Death Discov. 2022 Oct 31;8:435. doi: 10.1038/s41420-022-01217-9 (PMC9622744; doi:10.1038/s41420-022-01217-9)
Supplement: Supplementary file 1 — Full length WB Original Data [file 41420_2022_1217_MOESM1_ESM.pdf]

Full length WB (Figure 1)

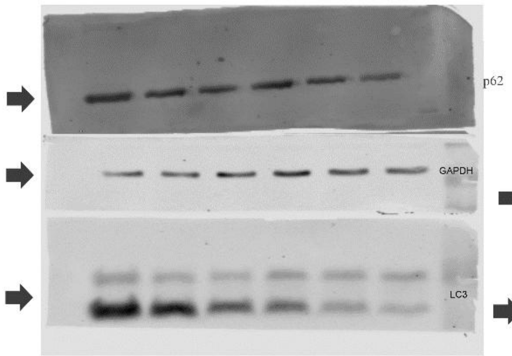

Figure 1C left

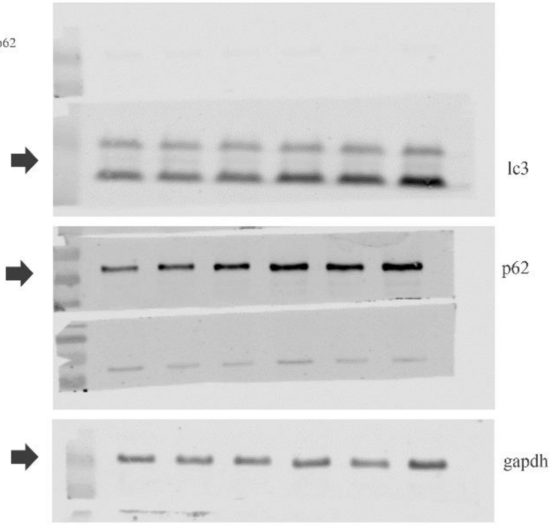

Figure 1C right

Full length WB (Figure 2)

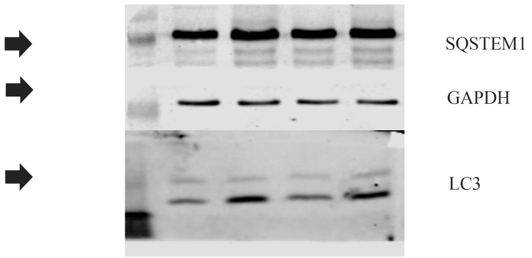

Figure 2 A

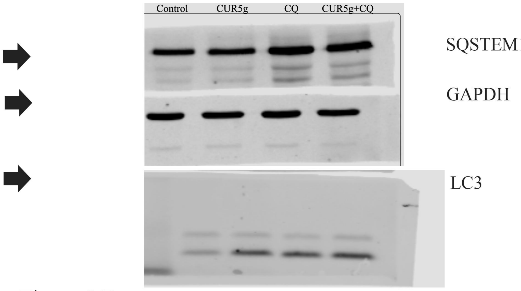

Figure 2 B

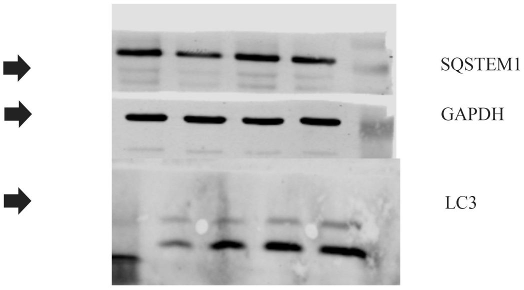

Figure 2 C

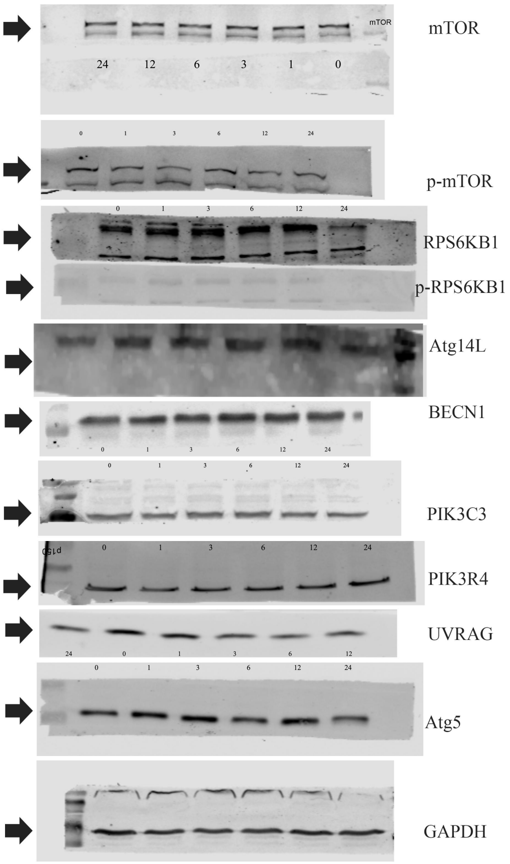

Figure 2 E

Full length WB (Figure 3)

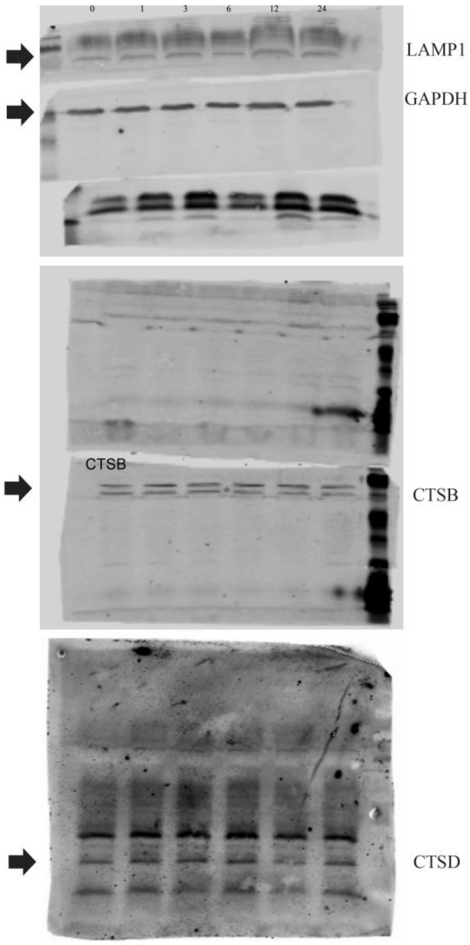

Figure 3 E

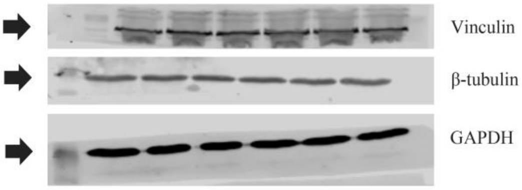

Figure 3 G

Full length WB (Figure 4)

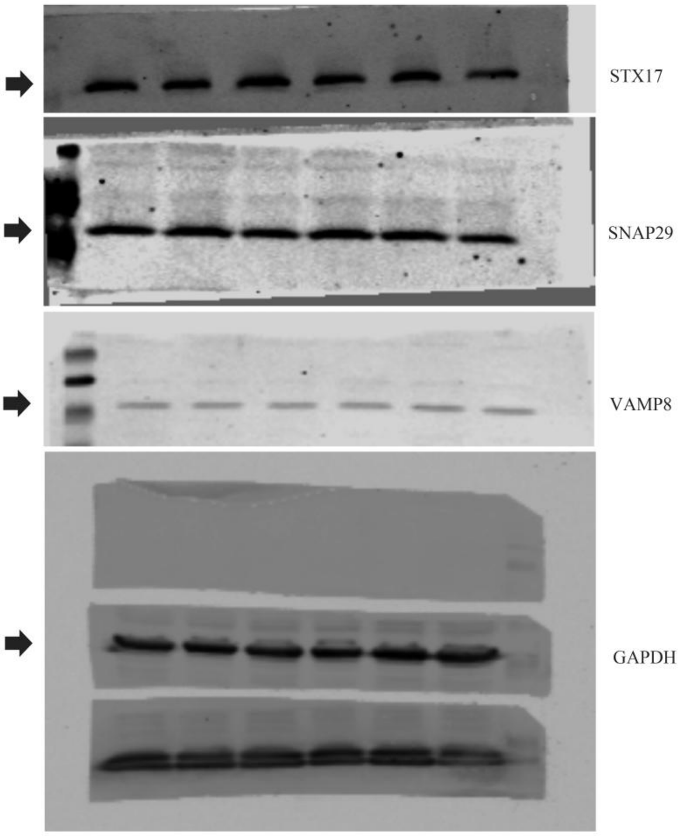

Figure 4 A

Full length WB (Figure 5)

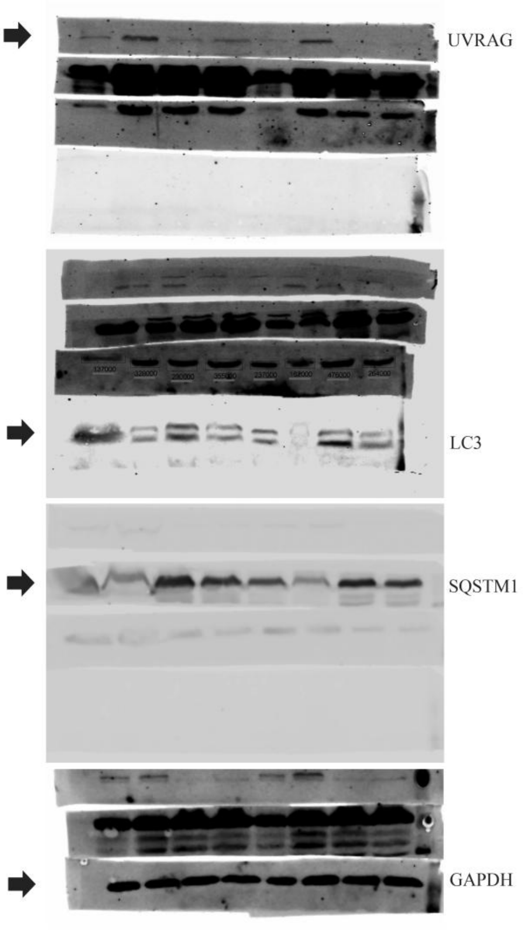

Figure 5 A

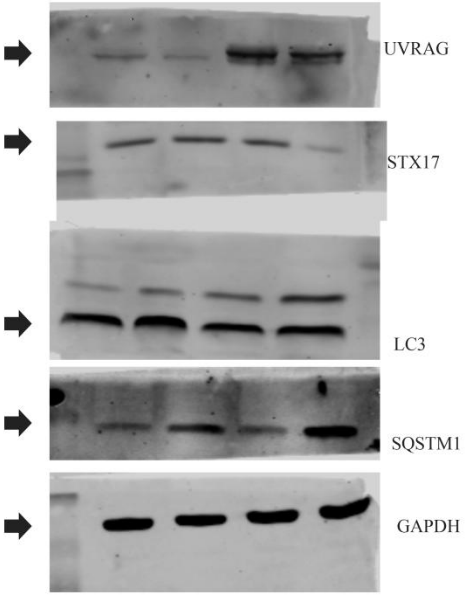

Figure 5 D

Full length WB (Figure 6)

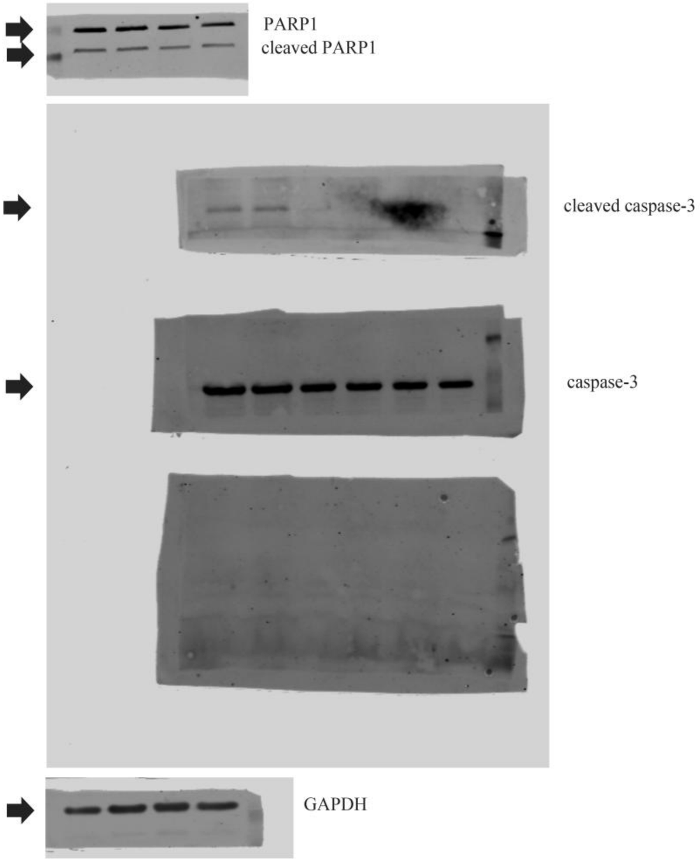

Figure 6 G

Full length WB (Figure 7)

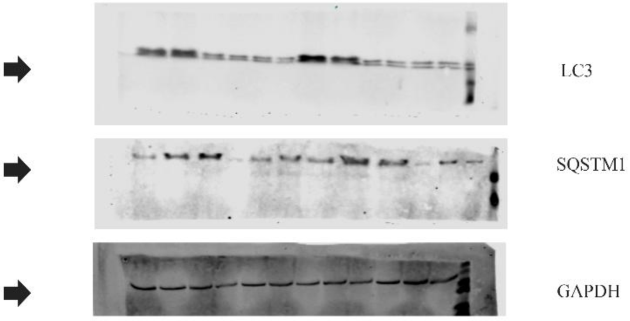

Figure 7 G

Full length WB (Figure S1)

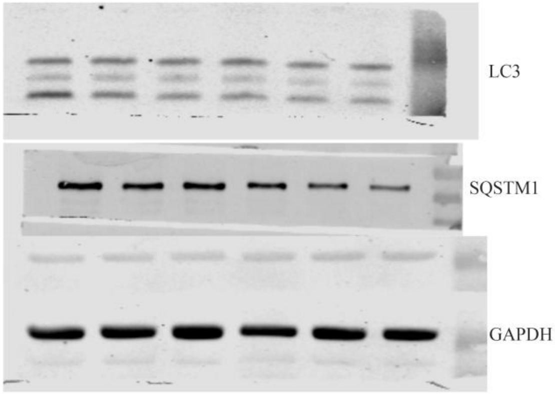

Figure S1 A

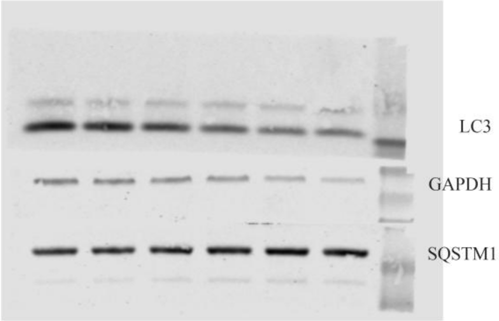

Figure S1 B

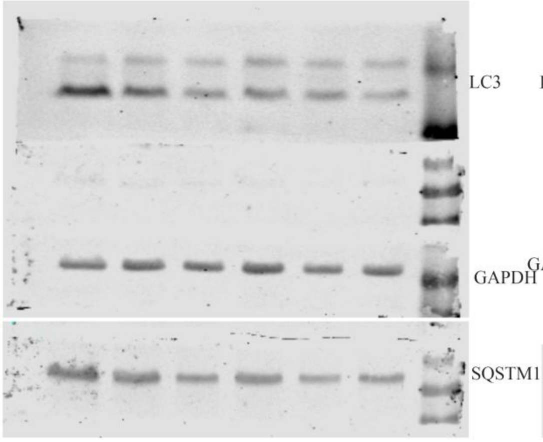

Figure S1 C

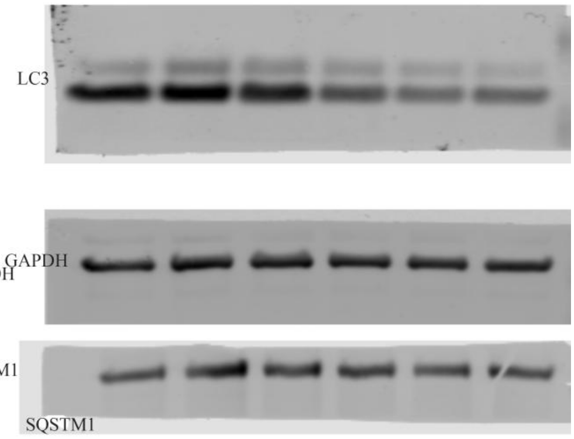

Figure S1 D

Full length WB (Figure S2)

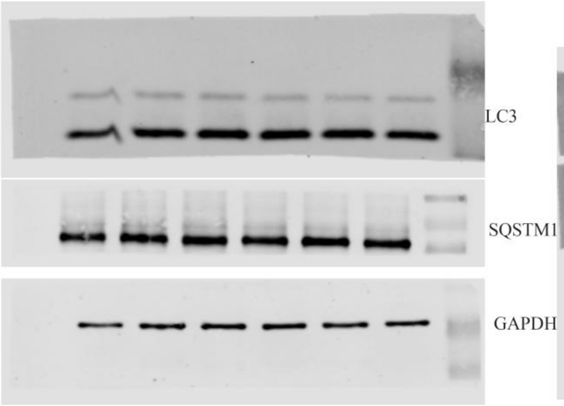

Figure S2 A

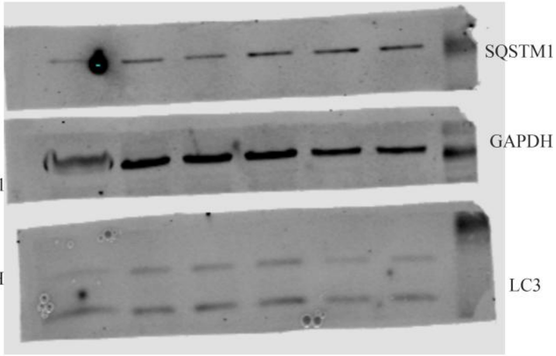

Figure S2 B

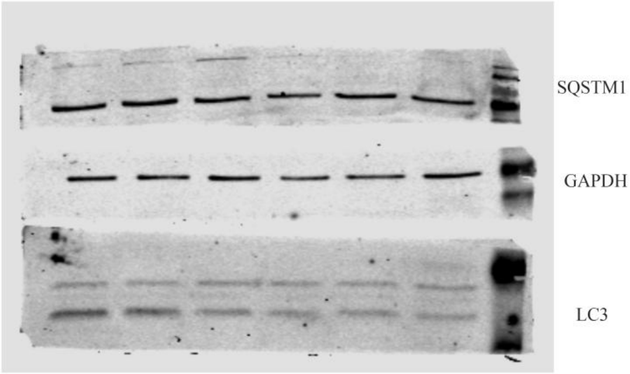

Figure S2 C

Full length WB (Figure S3)

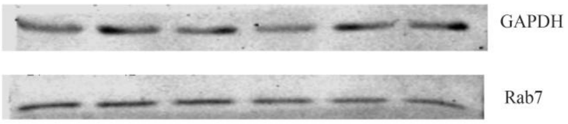

Figure S3 A

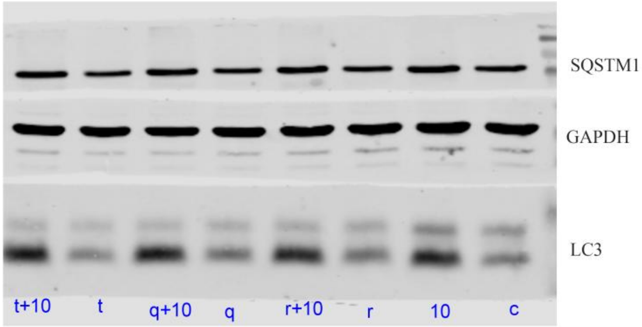

Figure S3 B

Full length WB (Figure S6)

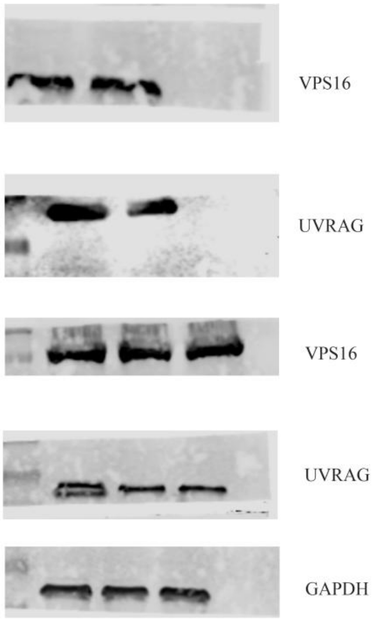

Figure S6 B

Full length WB (Figure S8)

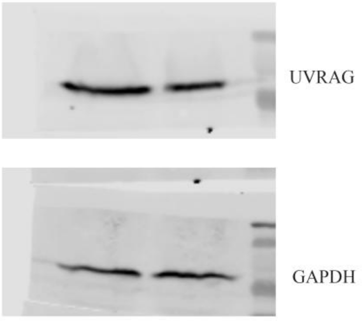

Figure S8 B

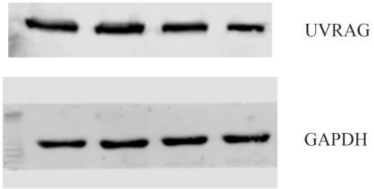

Figure S8 C
