# Supplementary material for: CUR5g, a novel autophagy inhibitor, exhibits potent synergistic anticancer effects with cisplatin against non-small-cell lung cancer
Source: Cell Death Discov. 2022 Oct 31;8:435. doi: 10.1038/s41420-022-01217-9 (PMC9622744; doi:10.1038/s41420-022-01217-9)
Supplement: Supplementary file 2 — Supplementary information-re [file 41420_2022_1217_MOESM2_ESM.docx]

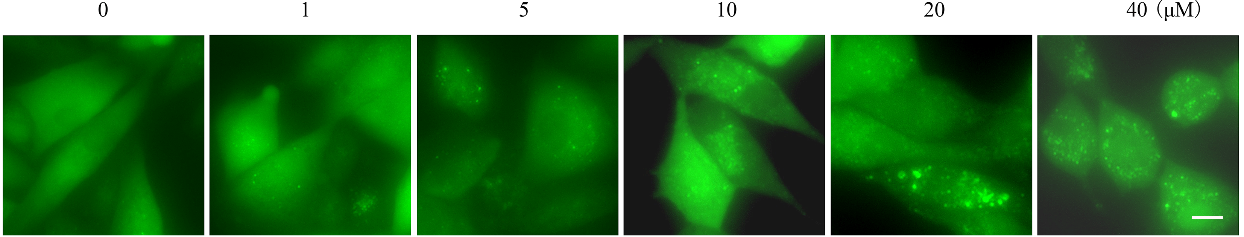


**Fig. S1 CUR5g induces a shift in GFP-LC3B signal from a diffuse cytosolic staining to a punctate pattern.** Representative fluorescence images of GFP-LC3B puncta in U87 cells treated with various doses (0-40 μM) of CUR5g for 24 h. Scale bar = 10 μm.

**
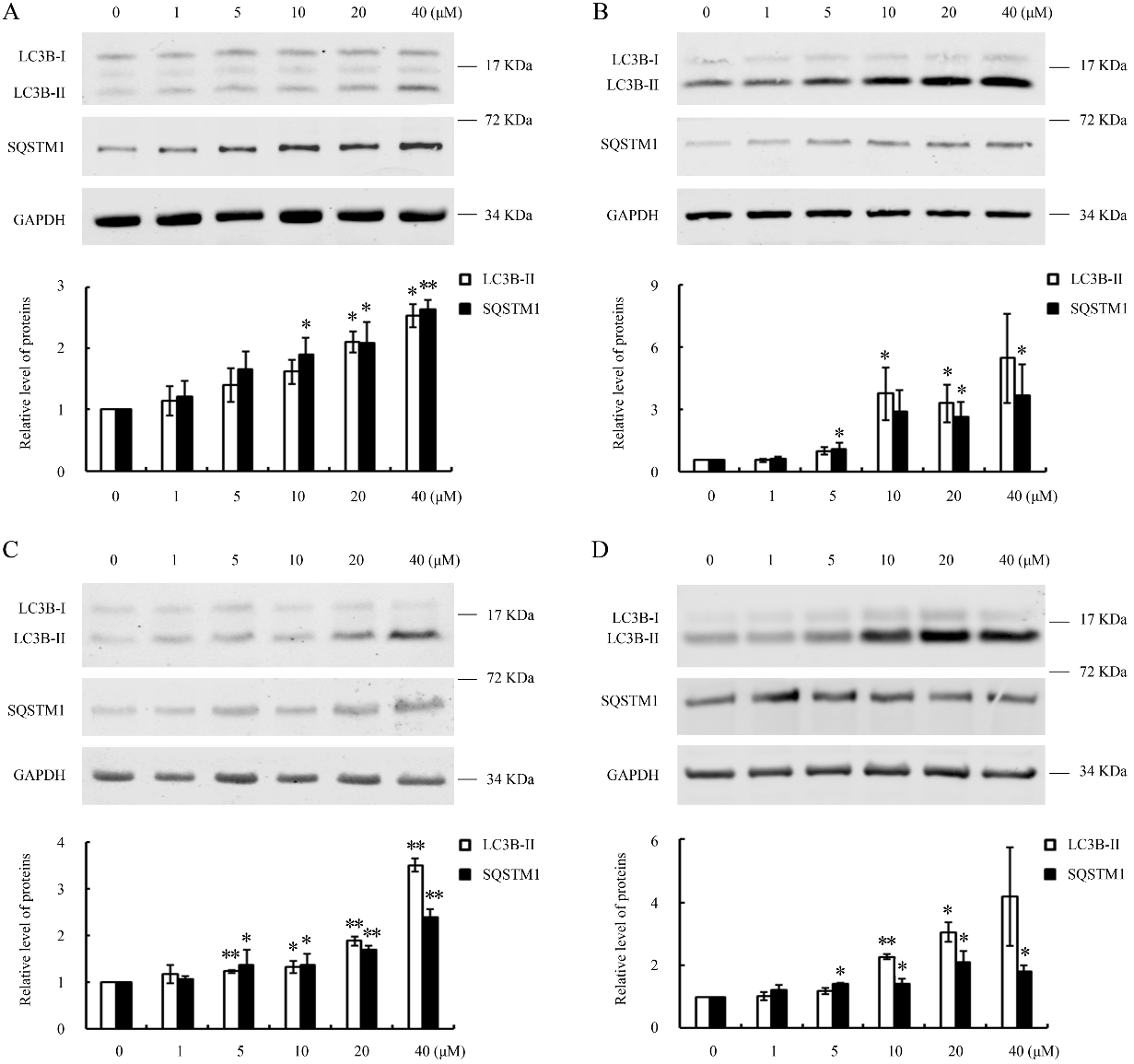
**

**Fig. S2 Western blot analysis of LC3B-II and SQSTM1 levels in cancer cell lines.** H157 cells (A), HepG2 cells(B), MCF-7 cells (C), and MCF-7/ADR cells (D) treated with various doses (0-40 μM) of CUR5g for 24 h. GAPDH is used as a loading control.

**
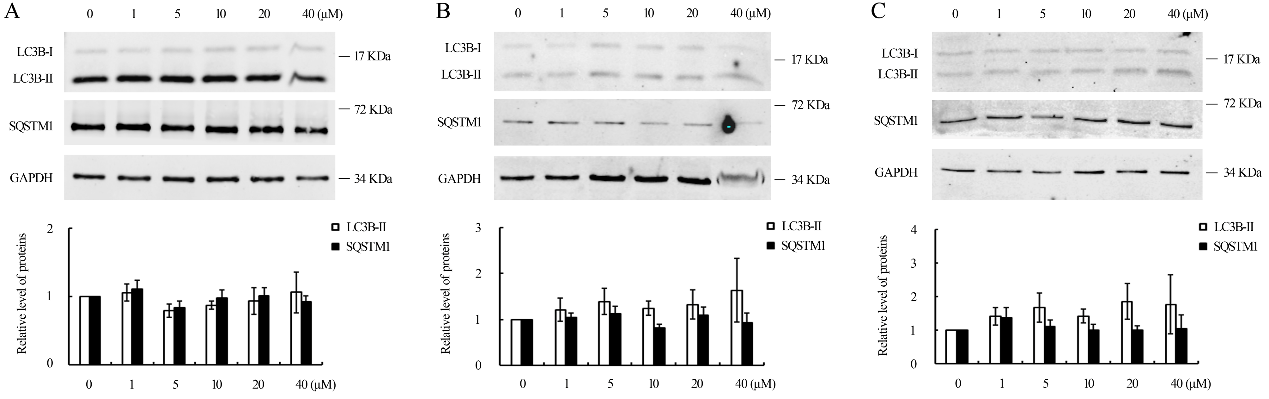
**

**Fig. S3 Western blot analysis of LC3B-II and SQSTM1 levels in normal cells.** BEAS-2B cells (A), HUVECs (B), and HBMECs (C) treated with various doses (0-40 μM) of CUR5g for 24 h. GAPDH is used as a loading control.

**
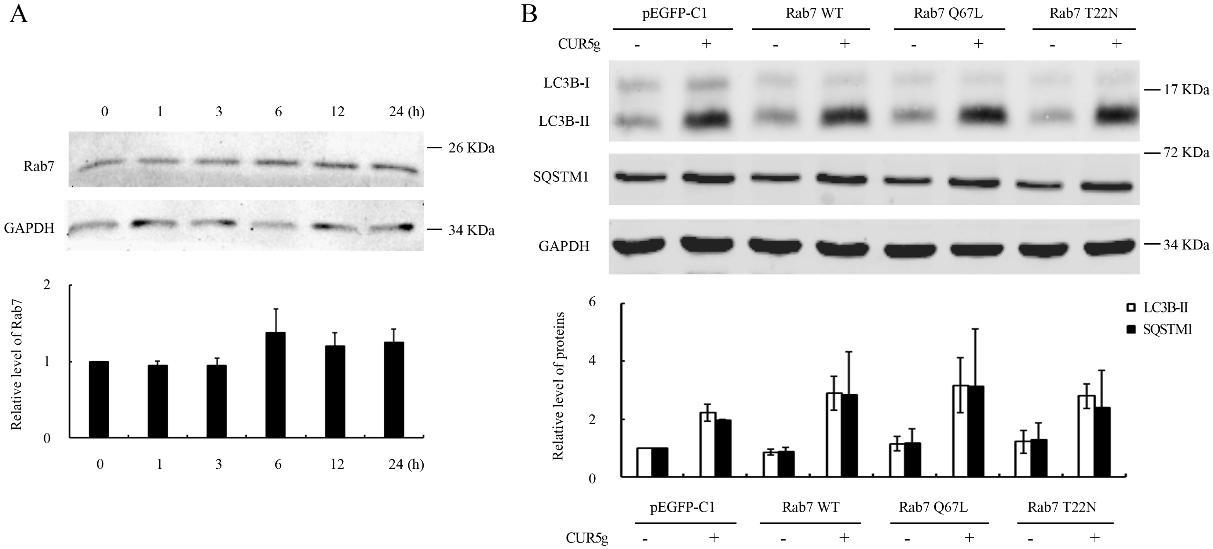
**

**Fig. S4 CUR5g blocks autophagy in a Rab7-independent manner.** A Western blot analysis of Rab7 levels in A549 cells treated with CUR5g (10 μM) for 0-24 h. GAPDH is used as a loading control. B Western blot analysis of LC3B-II and SQSTM1 levels in A549 cells transfected with wild-type pEGFP-Rab7 or the mutants Q67L or T22N in the presence of DMSO or CUR5g (10 μM) for 24 h.

**
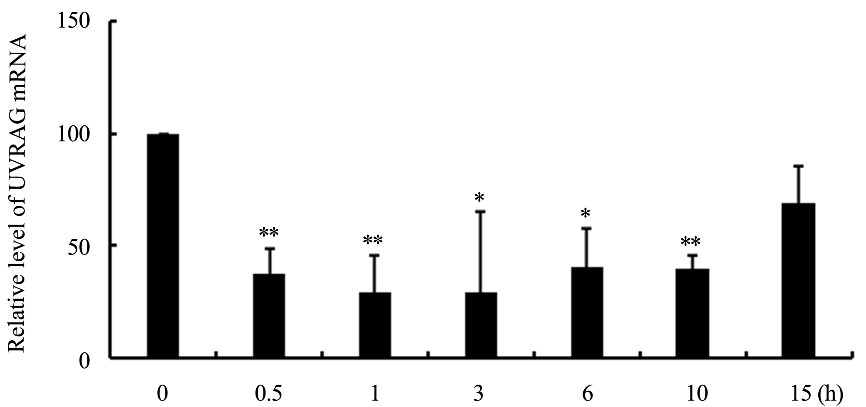
**

**Fig. S5 CUR5g decreases UVRAG mRNA level in A549 cells.** qRT-PCR analysis of UVRAG mRNA level in A549 cells treated with CUR5g (10 μM) for 0-15 h.


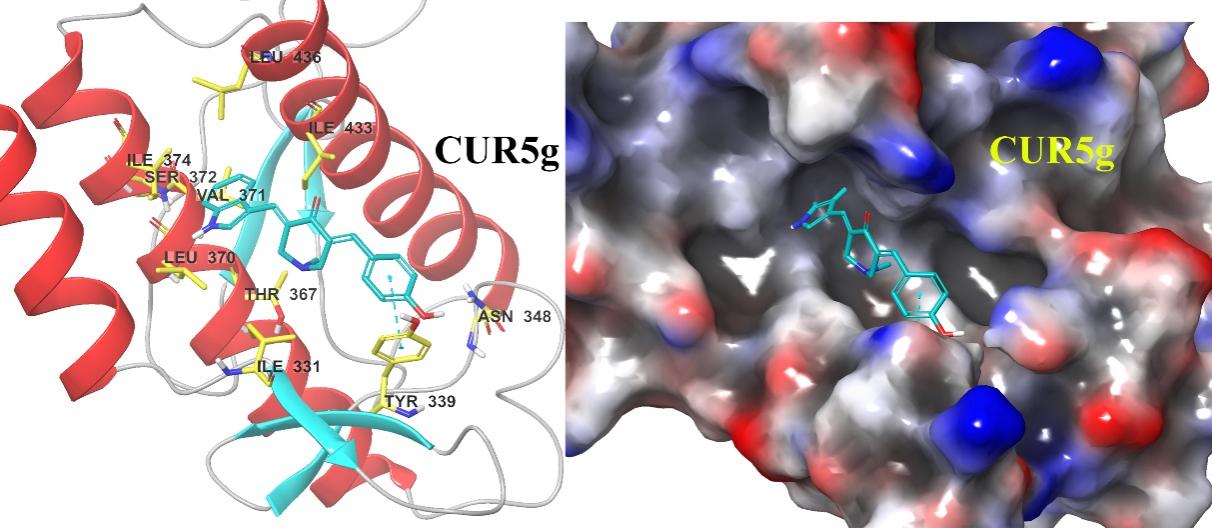


**Fig. S6 The binding interaction details of CUR5g with key amino acid residues on the best model of UVRAG.** The backbone structure and molecular surface of the UVRAG model bound with the computed most favorable conformation of CUR5g (carbon atoms in light blue). The negative-charged surface is indicated as red and positive charged as blue. The binding interaction details of CUR5g with key amino acid residues (carbon atoms in yellow), pi-pi stacking (light blue) is depicted with dashes.

**
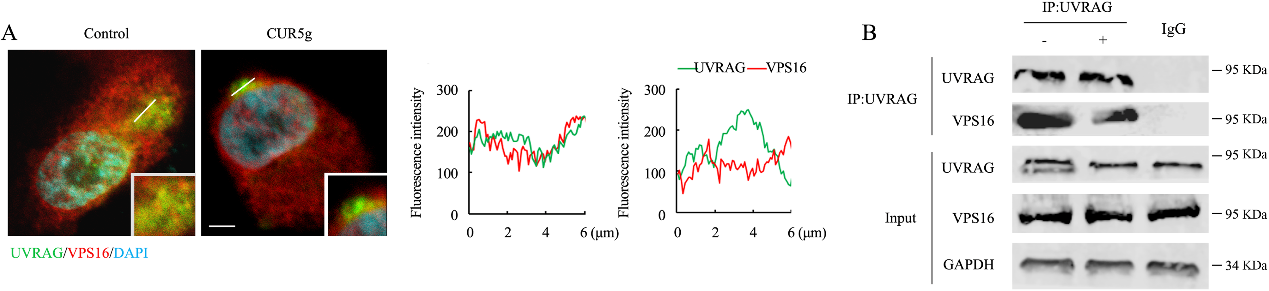
**

**Fig. S7 CUR5g weakens the interaction between UVRAG and VPS16.** A Representative fluorescence images of the colocalization of UVRAG (green) and VPS16 (red). Nuclei were stained with DAPI. The line scanned proﬁles show the distribution of ﬂuorescence for each channel in the white line in the corresponding confocal images. Scale bar = 5 μm. B The extracts of A549 cells were immunoprecipitated with anti-UVRAG antibody and analyzed by western blot with anti-UVRAG and anti-VPS16 antibodies.


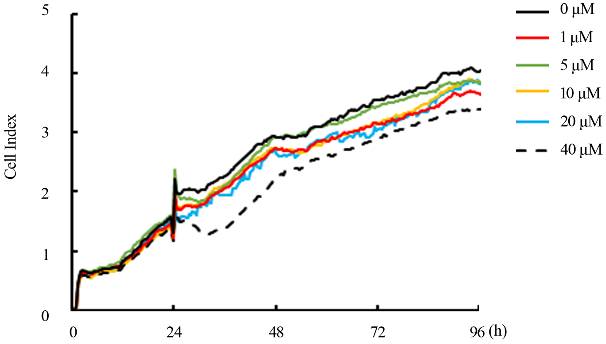


**Fig. S8 Effects of CUR5g on the cell viability of normal HUVECs.** Cell number was monitored over 96 h in a real-time manner using an xCELLigence RTCA S16 System. Cell-sensor impedance is displayed as the cell index. CUR5g (0-40 μM) was added after the cells were seeded into the plates for 24 h.

**
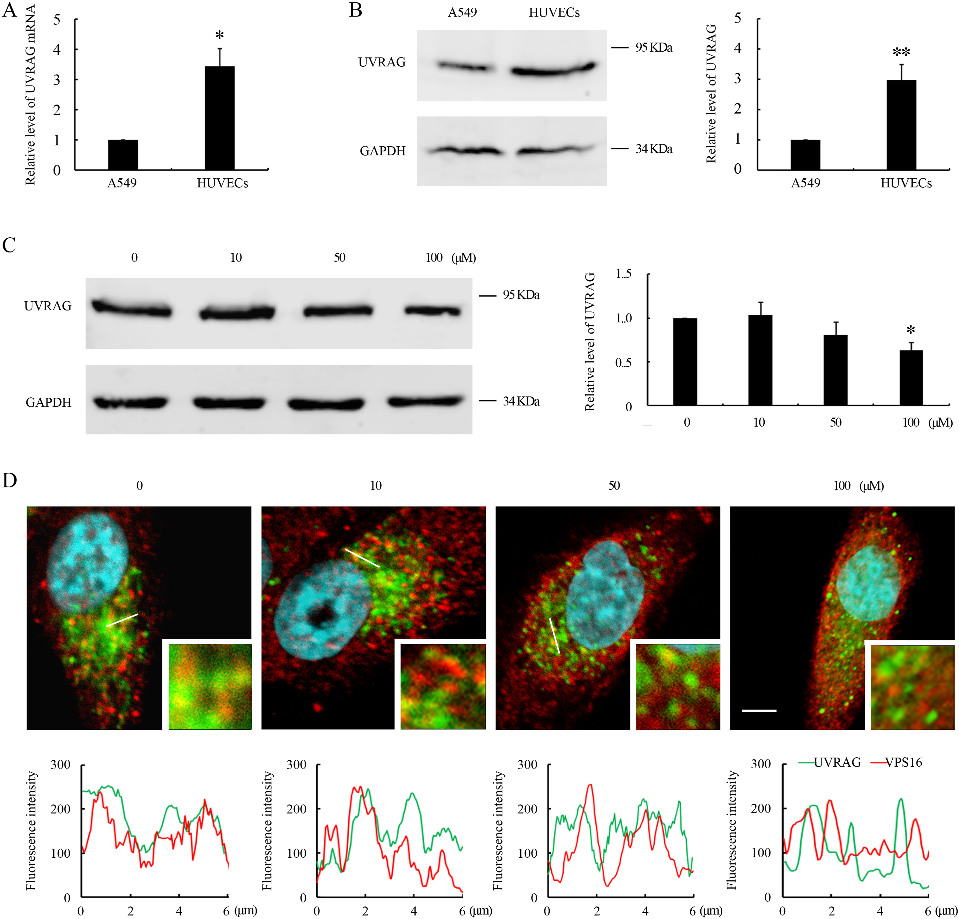
**

**Fig. S9 Effects of CUR5g on the level of UVRAG and the UVRAG-VPS16 interaction in normal HUVECs. A** qRT-PCR analysis of UVRAG mRNA levels in A549 cells and HUVECs. B Western blot analysis of UVRAG levels in A549 cells and HUVECs. C Western blot analysis of UVRAG levels in HUVECs treated with various doses (0-100 μM) of CUR5g for 24 h. D Representative fluorescence images of the colocalization of UVRAG (green) and VPS16 (red). Nuclei were stained with DAPI. The line scanned proﬁles show the distribution of ﬂuorescence for each channel in the white line in the corresponding confocal images. Scale bar = 5 μm.

**
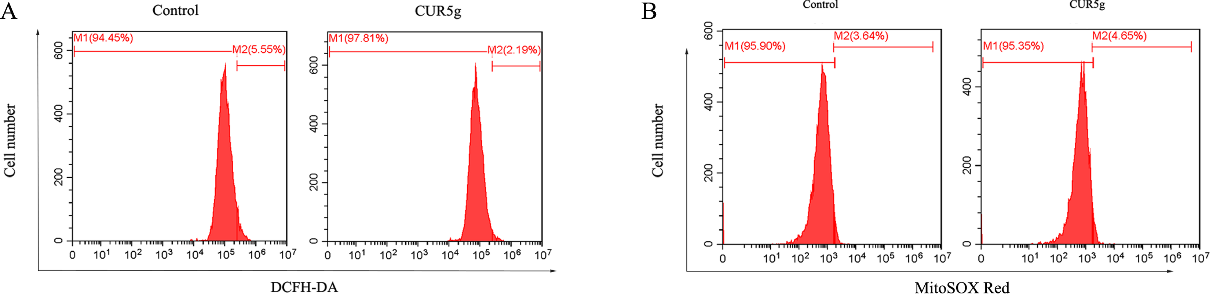
**

**Fig. S10 Effects of CUR5g on the intracellular ROS and mitochondrial ROS.** A549 cells were treated with DMSO or CA-5f (10 μM) for 24 h. A For determination of the intracellular ROS levels, the cells were loaded with 10 µM DCFH-DA for 20 min and collected for flow cytometry analysis. B To measure the mitochondrial ROS production, the cells were loaded with 5 µM MitoSOX® Red for 10 min and collected for flow cytometry analysis.

**
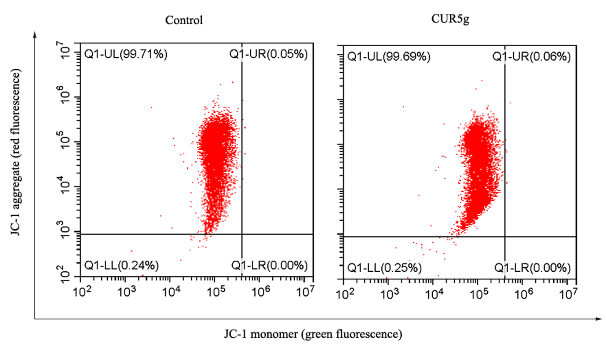
**

**Fig. S11 Effects of CUR5g on the mitochondrial membrane potential (MMP).** Fluorescence photographs of JC-1 staining in A549 cells treated with DMSO or CA-5f (10 μM) for 24 h. For determination of the MMP, cells were incubated with 10 µg/ml JC-1 for 20 min and collected for flow cytometry analysis.


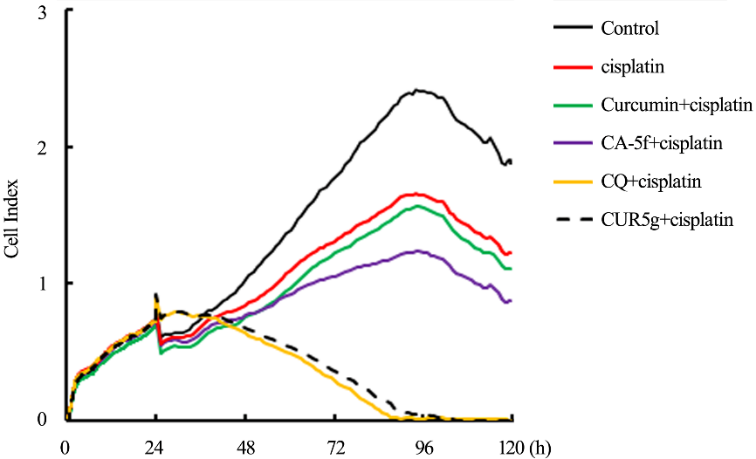


**Fig. S12 Mouse body weight. Comparison of efficacy of CUR5g combined cisplatin and other established autophagy regulators combined cisplatin.** Cell number was monitored over 120 h in a real-time manner using an xCELLigence RTCA S16 System. DMSO, cisplatin (30 μM), curcumin (10 μM) plus cisplatin (30 μM), CA-5f (10 μM) plus cisplatin (30 μM), CQ (30 μM) plus cisplatin (30 μM) or CUR5g (10 μM) plus cisplatin (30 μM) was added after the cells were seeded into the plates for 24 h.

**
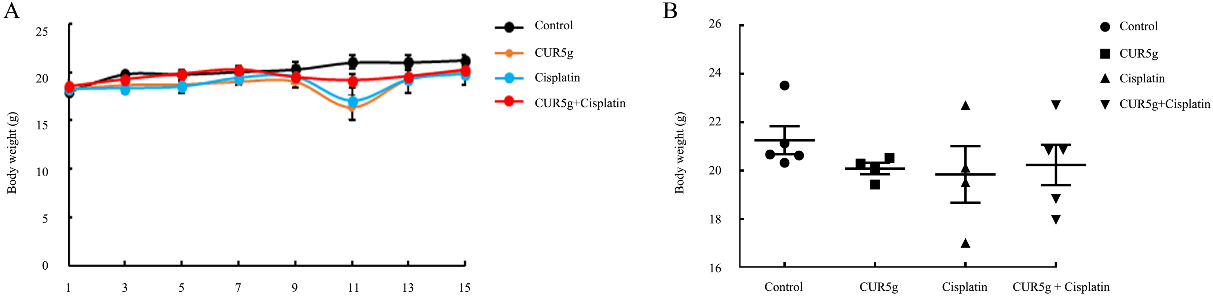
**

**Fig. S13 Mouse body weight.** A The mean body weight of each group was recorded every 2 days for up to 15 days. B The body weight of each mouse was recorded at the end of experiments.


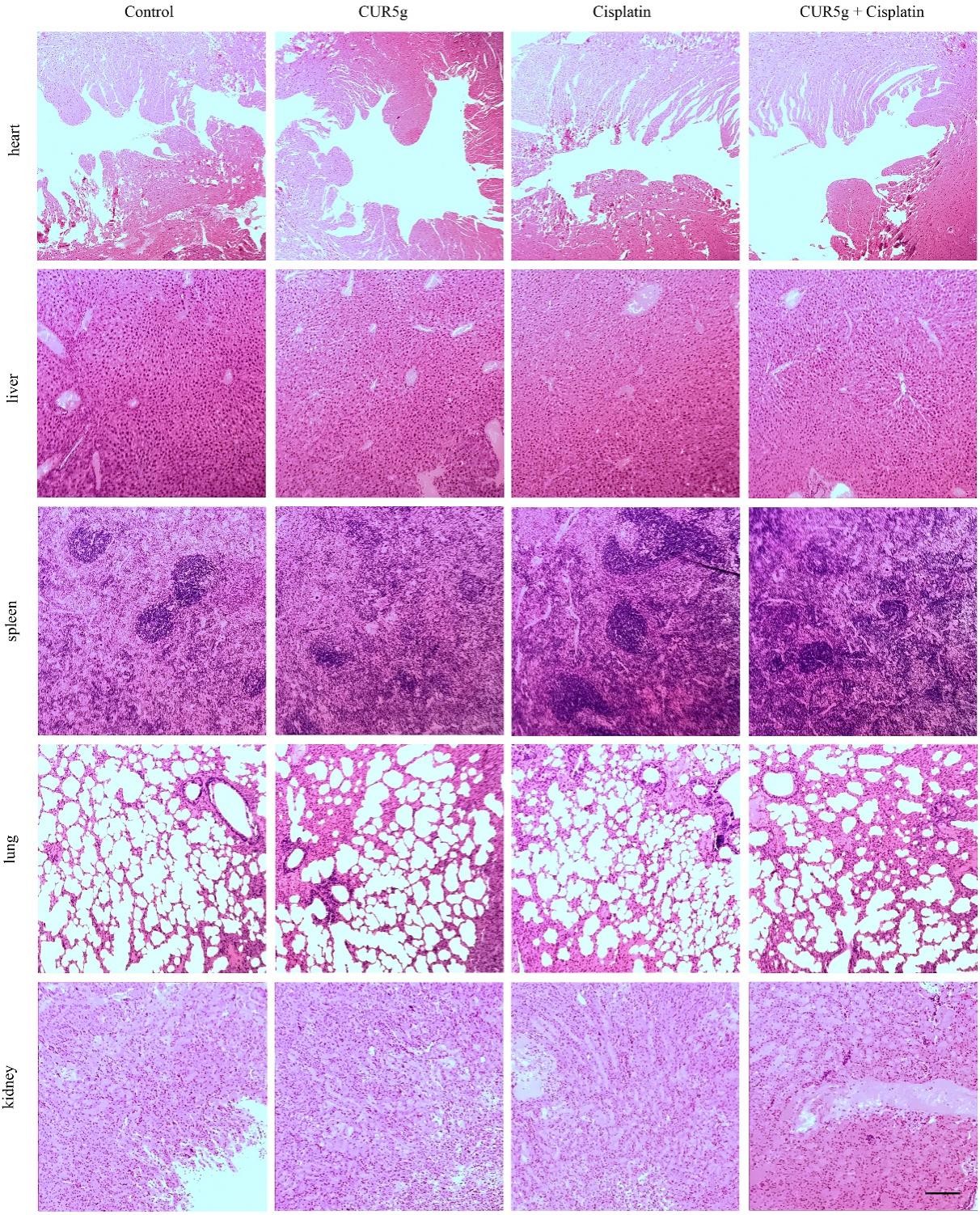


**Fig. S14 All animals did not exhibit abnormal histopathological changes.** Representative images of the histological examination of HE-stained sections of hearts, livers, spleen, lung and kidneys. Scale bar = 200 μm.
